# From nominalizer to verbalizer, from state to event: denominal verbs in -*euō* in early ancient Greek

**DOI:** 10.1007/s11525-026-09472-7

**Published:** 2026-10-20

**Authors:** Carolina Marescotti, Laura Grestenberger

**Affiliations:** Department of Linguistics, University of Vienna, Vienna, Austria

**Keywords:** Ancient Greek, Nominalizer/verbalizer, Reanalysis, Davidsonian/Kimian states, Distributed Morphology

## Abstract

This paper investigates the diachronic development of the Ancient Greek suffix -*eu*- from a nominal marker into a verbalizer, focusing on the transition of -*euō* verbs from stative to activity/eventive meanings. Building on Distributed Morphology and the Hale & Keyser/Harley framework of denominal verb formation, we argue that verbal -*eu*- emerged through the reanalysis of “deagentive” denominal verbs, i.e., verbs derived from agentive animate nouns. These verbs exhibit an inherent ambiguity between stative and eventive readings, which we analyze as reflecting Davidsonian state readings. Unlike Kimian states, Davidsonian states contain an event variable, making them compatible with event-related diagnostics such as manner adverbs, locative phrases, and perception verbs. This eventive potential provided the basis for a categorial shift whereby nominal -*eu*- was reinterpreted as an activity verbalizer. Corpus evidence from Homeric and early first-millennium BCE Greek shows that the earliest -*euō* verbs already patterned with Davidsonian states, suggesting that the reanalysis predates the Homeric period. We further argue that the morphosemantic properties of the suffix reflect those of its historically nominal base: the features [+animate, +agentive] of the base are transferred from the subject of the denominal verb to the verbal event itself, accounting for the animate, usually agentive subjects of early -*euō* verbs. This development illustrates how root-adjacent morphology can give rise to new verbalizers, reinforcing models in which the linear order of morphemes mirrors syntactic structure. It also contributes to the cross-linguistic understanding of the relationship between stativity, eventivity, and verbal derivation.

## Introduction

The theoretical status of “root-adjacent” — stem-forming or *categorizing* — verbal morphology and its interaction with argument and event structure alternations has recently become the subject of intense debate, especially concerning the function(s, if any) of verbal theme vowels and conjugational class markers (e.g., Oltra-Massuet, 1999; Oltra-Massuet & Arregi, 2005; Spyropoulos et al., 2015; Panagiotidis et al., 2017; Kastner & Martin, 2021; Simonović & Mišmaš, 2023; Kovačević et al., 2024), the status of verbal derivational morphemes as roots vs. as affixes (e.g., de Belder, 2011; Lowenstamm, 2014; Creemers et al., 2018; Cavirani-Pots, 2020), and the morphosemantics of non-concatenative categorizers such as verbal templates (e.g., Doron, 2003; Alexiadou & Doron, 2012; Kastner, 2020). However, these elements are rarely treated from a diachronic perspective, so there is an insufficient empirical basis for generalizations as to how such categorizers arise and whether and to what extent they become associated with morphosyntactic functions (such as transitivization, lexical aspect, voice) in the course of their development. Some recent studies that do tackle these elements from a diachronic perspective within the framework of Distributed Morphology include, e.g., Bertocci (2017), Grestenberger (2022, 2023), Calabrese (2023), Calabrese and Petrosino (2023).

In this article, we take this work as a starting point to argue that one way in which new verbalizers arise is through reanalysis of root-adjacent material in the context of cross-categorial derivation, and that the morphosemantic properties (e.g., phonological, selectional, and argument structure-related properties) of the new verbalizer follow from the morphosemantic properties of the diachronic derivational base in a *directional* fashion (Grestenberger, 2023). We argue that this provides evidence for theoretical models in which morphology mirrors syntax (understood here in terms of the linear order of components, but we will elaborate on this below), such as Distributed Morphology (DM) and more specifically for a bottom-up, inside-out approach to Vocabulary Insertion (e.g., Bobaljik, 2000; Embick, 2010, 2012; Paparounas, 2024). Specifically, the diachrony of verbal categorizers provides evidence that both the phonological *and* the semantic properties of nodes that are lower in the structure are reanalyzed as belonging to structurally adjacent higher nodes rather than, for example, skipping intermediate nodes or becoming associated with the stem or the word-level. We moreover argue that this development is *directional*, in that the reanalysis always proceeds from the lower to the higher node rather than vice versa. In terms of linear order of exponents this means that, assuming that the concatenated sequence *X*^⌢^*Y* in (1b) linearizes the complex head in (1a) (as standardly assumed in suffixing languages such as Greek), *Y* can inherit phonological and semantic features from *X*, but not vice versa (cf. Grestenberger, 2023; Calabrese & Grestenberger, 2024). Concretely, this predicts that in denominal verbs in languages like Greek, nominal suffixes can become verbalizers diachronically, but verbalizers cannot become reanalyzed as new nominal suffixes in such structures. This prediction significantly constrains the available diachronic “reanalysis space” in the domain of complex word formation.

**Figure:**
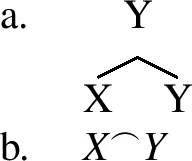

The case study we focus on here comes from Greek, whose long attestation history provides an ideal environment for tracking changes in categorizing morphology that span several centuries and even millennia.^1^ We discuss the development of the Ancient Greek (AG) suffix -*eu*-, an originally *nominal* derivational suffix that formed animate substantives and which turned into a *verbal* derivational suffix already during the classical period, and eventually into the Modern Greek (MG) verbalizer -*ev*-. This paper focuses on the starting point of this development, namely denominal “deagentive” verbs, which were historically derived from animate *nouns* in -*eu*-. In the literature, these verbs are frequently described as either expressing states (‘be X’) or events, specifically activities (‘act as/like X’), for example in Schwyzer (1939, 732). This apparent ambiguity has been noticed for “behavior-related” verbs more broadly (e.g., Tucker, 1990, 75ff.; Martin & Piñón, 2020) and raises the question how the apparent stative reading of such verbs differs from the corresponding copula constructions — in the case of Greek, for example, *aristeúō* ‘be the best’ vs. *áristos* ‘best’ or *aristeús* ‘best one’ + *eimì* ‘be’; *basileúō* ‘be king’ vs. *basileús eimì* ‘be king’, etc. Thus the difference between the use of the copula construction in (2)(*áristos* ‘best’ + copula) and the use of verbal *aristeúō* ‘be best’ in (3)is not immediately clear; note that the latter is even in a coordination structure with a copula construction in (3)(*hupeírokhos* ‘prominent, eminent’ + copula).^2^

(2)**Figure:**
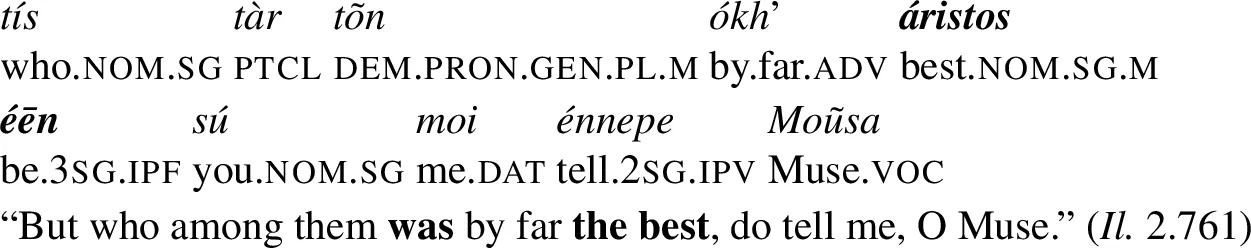

(3)**Figure:**
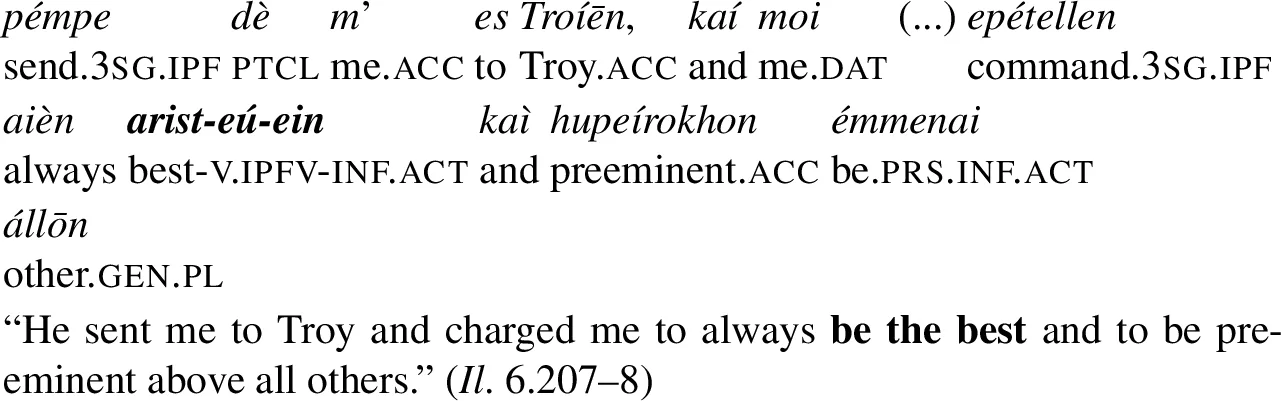

This is not merely a problem of translation, as the same apparent competition exists in contemporary languages (see Martin & Piñón, 2020). While we cannot address the properties of the copula strategy here for reasons of space, we argue that the properties of denominal deagentive or “behavior-related” verbs in Ancient Greek is in line with recent claims that these types of verbs are always stage level (SL) predicates, while the corresponding copula constructions can be individual level (IL) or SL predicates (Acedo-Matellán & Gibert-Sotelo, 2022; Acedo-Matellán, 2022; Acedo-Matellán & Oltra-Massuet, 2024). Verbalization, then, *always* introduces a particular type of event variable (a “Davidsonian” event, in our terminology, cf. Sect. 5), and it is this event variable, we argue, that provided the starting point for the reanalysis of nominal -*eu*- as a verbalizer in the context of deagentive denominal verb formation. Drawing on a corpus study of the earliest attestations of denominal deagentive -*euō* verbs in Greek, that is, the Homeric stage, which represents the earliest attested phase of Ancient Greek (early 8th century BCE), the paper argues that the suffix inherited a specific set of functions from its animate nominal base that determined the semantics of the derived verbs as well as of the new reanalyzed verbalizer that emerged from them, namely, as forming Davidsonian events with animate subjects. From this stage, the resulting verbalizer formed intransitive activity verbs and subsequently also transitive activity and accomplishment verbs, eventually developing into an across-the-board verbalizer.

This article is structured as follows. In Sect. 2, we introduce the theoretical underpinnings of our analysis, specifically the status of categorizing morphology in Distributed Morphology (DM), Sect. 2.1, and the Hale & Keyser/Harley-approach to denominal verb formation and its implications for the diachrony of verbalizing morphology (Sect. 2.2). Section 3 provides the general background on verbal -*eu*- in Ancient and Modern Greek and on the *nominal* uses of -*eu*- in Ancient Greek. Section 4 presents the data in our corpus of Ancient Greek -*euō* verbs and the semantic classification of these verbs (Sect. 4.3). Section 5 discusses the properties of the deagentive class of -*euō* verbs with respect to the distinction between the stative and the eventive reading and argues that deagentive -*euō* verbs have a “Davidsonian state” reading already at the Homeric stage. We also discuss the further development of these verbs into transitive activity and accomplishment verbs (Sect. 5.3). In Sect. 6, we present our analysis of deagentive -*euō* verbs as obligatorily eventive and of the reanalysis of nominal -*eu*- as a verbalizer in this context. Section 7 contains the conclusion.

## Background

### Categorizing morphology in Distributed Morphology

Among the core tenets of DM is the principle of Full Decomposition: *All* word forms can be decomposed into component parts, consisting of a root and functional (terminal) nodes (Halle & Marantz, 1993; Harley & Noyer, 1999; Embick & Noyer, 2007; Embick, 2015; Bobaljik, 2017). These roots and nodes are then spelled out or “realized” depending on their feature content and context. Because roots themselves are categorially unspecified, they must initially combine (“merge”) with a *categorizing* functional head: a verbalizer *v*, nominalizer *n* or adjectivizer *a*, as illustrated in (4)(e.g., Embick, 2015; Alexiadou & Lohndal, 2017). Once categorized, the resulting complex head then merges with higher functional projections such as Voice, Asp(ect) and T(ense) in the case of *v*. In complex word forms — verb forms, in our case — the root then moves upwards and forms a complex head with *v* (i.e., [*v* [$\sqrt{}$ *v*]]), which in turn moves upwards and forms a complex head with Voice, Asp, and so on. The resulting complex head is then realized through linearly ordered Vocabulary Items by Vocabulary Insertion.

(4)**Figure:**
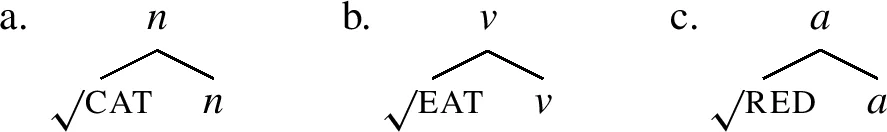

In DM, phonologically null or “zero” categorizers are posited for examples like (4)in English,^3^ though in Ancient (and Modern) Greek, (verbal) categorization is usually overt. We moreover follow much of the literature in assuming that these categorizers can be associated with specific morphosemantic functions or “flavors” (on *v*, e.g., Folli & Harley, 2005, 2007; Harley, 2005, 2009, 2013a; Alexiadou & Lohndal, 2017; Panagiotidis et al., 2017; Kovačević et al., 2024). Like other categorizers, *v* mediates between the root and higher functional projections. Argument and event structure alternations such as the causative alternation, passivization, etc., are due to the interaction of *v* with the external-argument introducing projection Voice (Alexiadou, 2013; Harley, 2013a, 2017; Alexiadou et al., 2015; Schäfer, 2008, 2017, Kastner, 2020; Grestenberger, 2021b, 2022). Crucially, the order of morphemes in a complex word form is expected to *mirror* the order of functional projections built up by the syntactic component (the Mirror Principle, Baker, 1985) because of the “inside-out” nature of Vocabulary Insertion, which starts from the root and proceeds outwards. In terms of linear order, the expected surface order of exponents associated with the abstract concatenated sequence (5a) is thus (5b).

(5)**Figure:**
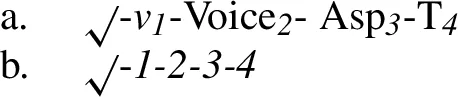

Pruning of phonologically empty nodes (e.g., Embick, 2010; Christopoulos & Petrosino, 2018; Calabrese & Petrosino, 2023; Paparounas, 2024), Fusion, and postsyntactic reordering operations such as Local Dislocation and Lowering (Embick & Noyer, 2001; Harley, 2013b; Embick, 2015) before Vocabulary Insertion can create language-specific variation in the actually attested surface order of morphemes. Moreover, allomorphy and the competition for insertion of exponents further complicate the picture.^4^ What is important for our purposes is that the underlying order of functional projections and hence the expected surface order of exponents (and deviations from it) need to be independently motivated, and it is crucially the surface linear order that will be relevant for our analysis in Sect. 6. For the Ancient Greek verbal system, we assume based on previous work (e.g., Grestenberger, 2021b, 2022; Schreiner, 2021; Calabrese & Petrosino, 2023) that the surface realization of terminal nodes is as follows (leaving aside the realization of Mood):

(6)**Figure:**
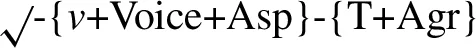

*v*+Voice+Asp and T+Agr represent fused nodes that are morphologically expressed by verbal stem-forming and inflectional agreement morphology, respectively.^5^ In denominal verbs, the structure is therefore as follows:

(7)**Figure:**
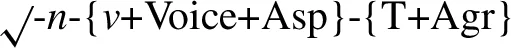

There is a long tradition of research that argues that in contexts of cross-categorial derivation like (7), the syntactic and semantic features of the nominal base have an effect on the way the derived verb behaves with respect to Aktionsart (actionality), argument structure, and voice, though the nature of the relevant features (e.g., ±bounded, mass/count, etc.) and the extent to which they determine the behavior of the resulting verb are open to discussion (Clark & Clark, 1979; Kiparsky, 1997; Harley, 1999, 2005; Bleotu, 2019; Grestenberger & Kastner, 2022; on Ancient Greek in particular see Tucker, 1990; Barber, 2005, 2013; Marescotti, 2024, 2026). In Sect. 6, we will argue that the AG nominal “pseudo-agent” suffix -*eu*- became reanalyzed as part of the linearly adjacent, structurally “higher” verbalizer *v* in structures like (7)and that this reanalysis resulted in a particular morphosemantic “flavor” of *v* in these verbs. In the next section, we show that this is predicted by the decompositional analysis of unergative activity and accomplishment verbs of Harley (2005).

### Denominal verbs and Aktionsart: Harley (2005)

Apart from the type of base, different types of verbalizers are also assumed to interact with cross-categorial derivation contexts in different ways. According to Harley (2005, 2011), building on work by Hale and Keyser (e.g., Hale & Keyser, 1998, 2002), the verbalizer *v*_do_ creates unergative verbs that are essentially denominals in which a bare noun^6^ incorporates into or “conflates with” the selecting verbal projection *v*_do_. Unaccusatives/change-of-state verbs (the anti-causative members of the causative alternation), on the other hand, are syntactically deadjectival verbs in this approach, in that an adjective incorporates into/conflates with *v*_become_. (8)illustrates the proposed structure of unergatives and unaccusatives according to this proposal.

(8)**Figure:**
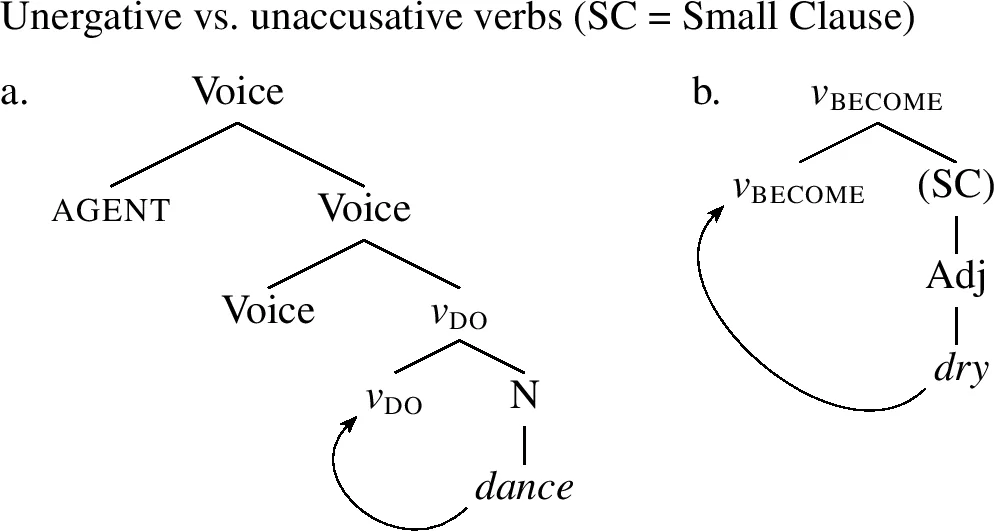

The structure in (8a) is the one that is relevant for our purposes: basically, what Hale & Keyser and Harley argue is that unergative verbs like *laugh*, *dance*, *run*, and *hop* are underlyingly “laugh-do’, ‘*dance*-do’, etc., based on the cross-linguistic comparison with languages in which unergatives such as these are expressed via light verb constructions with do and a nominal expressing the event. Moreover, Harley notes that different types of roots, namely things vs. events, bounded vs. unbounded bases, give rise to differences in the behavior of unergatives with respect to telicity and durativity (see (9), (10). In other words, the Aktionsart and telicity of verbs like those in (8)is compositionally predictable from 1) the type of selecting verbalizer (*v*_do_, *v*_become_, etc.), 2) the type of element that conflates with the selecting verbalizer (e.g., whether it is ±bounded or an event vs. thing vs. state root, etc.) and 3) whether or not the element that conflates takes a complement. The examples in (9)illustrate this for denominal unergative accomplishments vs. activities in English. While verbs that are based on [+bounded] thing roots give rise to telic readings according to standard tests, (9a), verbs that are based on [-bounded] thing roots give rise to atelic verbs, (9b) (see also Marescotti, 2026, 8-12).

(9)**Figure:**
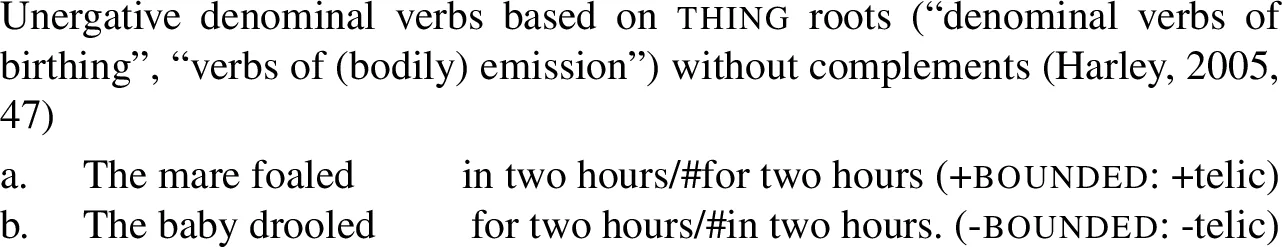

The examples in (10)show that [-bounded] event roots form atelic unergative activities (such as *dance*), (10a-b), while [+bounded] event roots (such as *hop*, *trip*) form semelfactives expressing instantaneous events (although they can be coerced into an activity reading via iteration), (10c-d).

(10)**Figure:**
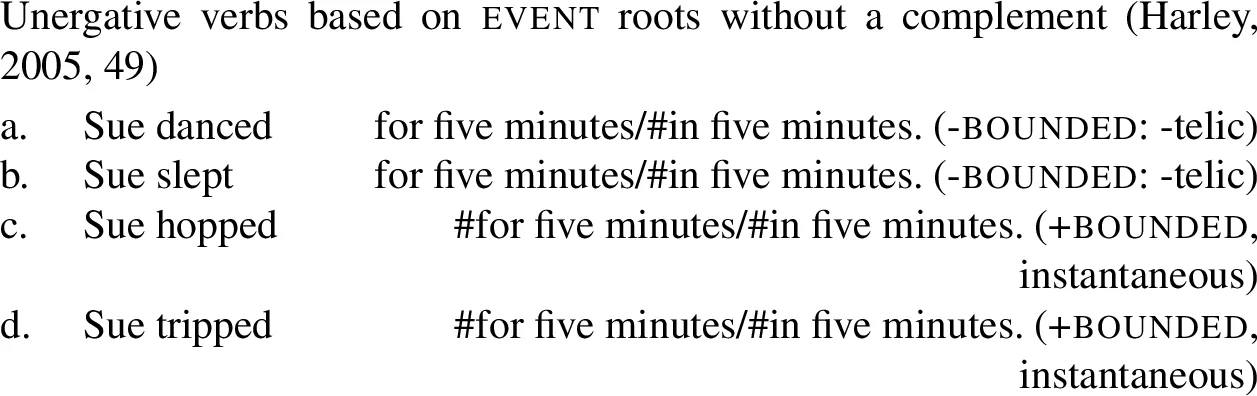

We forgo a discussion of the other verb classes in Harley (2005) here for reasons of space. What is crucial for our purposes is that the structure of verbs such as the ones in (9)–(10)is underlyingly the same, (11). Differences in telicity follow from differences in the (abstract) semantic properties of the conflating element, which is a root in Harley’s approach (a “thing root” for unergative accomplishments and an “event root” for unergative activities), but which, as we will see in Sect. 3, can also be an overtly categorized noun in Ancient Greek.

(11)**Figure:**
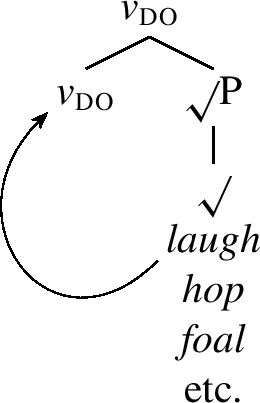

Some interesting diachronic predictions concerning the relationship between verbalizing morphology and argument structure follow from the Hale & Keyser/Harley approach. First, if new verbalizers arise through directional reanalysis in cross-categorial derivation, as argued in Sect. 6, then the abstract properties of the reanalyzed *n* or *a* should be systematically reflected in the abstract properties of the resulting verbalizer at least initially. This follows from the *synchronic* analysis proposed by Hale and Keyser (1998, 2002) and Harley (1999, 2005), in which features such as [±bounded] of the selected element and its ability to take a complement determine the Aktionsart of the derived verb. If nouns with similar properties marked with the same *n* (for example, specific agent nouns or verbal abstracts) become the input to denominal verb formation and are subsequently reanalyzed, then their properties should be systematically reflected in the event and argument structure properties of the resulting verbalizers (see Grestenberger, 2023 for a more detailed discussion of these predictions). In the following sections, we show that this is precisely what we see in the diachrony of the Greek verbalizer -*eu*-.

## The suffix -*eu*-/-*ev*- in Ancient and Modern Greek

### Overview: word-formation properties of -*eu*-/-*ev*-

Modern Greek has a number of productive verbalizers that historically go back to *nominal* suffixes, for example *-en-*, -*iz-*, -*ar-*, -*on-*, -*(i)az-*, and -*ev-* (Ralli, 2005; Efthymiou, 2011; Efthymiou et al., 2012; Holton et al., 2012; Spyropoulos et al., 2015; Panagiotidis et al., 2017; Koutsoukos, 2021, etc.). The verbalizer -*ev-* is moderately productive in Modern Greek and forms stative, inchoative, activity, and accomplishment verbs from nouns, adjectives, and adverbs (Efthymiou et al., 2012). It is also used to nativize loanwords. Examples of these functions are given in Table 1. In the Greek varieties spoken in Italy (Griko/Grecanico), it is moreover the predominant derivational suffix used to verbalize Romance stems and English loanwords (Losavio, 2024).

**Table 1:** Modern Greek verbs in -*ev*- (ex. from Panagiotidis et al., 2017)

MG *-ev-o*		Base		
*stox-****év****-o*	‘I aim at’	*stóx-os*	‘target’	noun
*fronim-****év****-o*	‘I become prudent’	*frónim-os*	‘prudent’	adj.
*kont-****év****-o*	‘I approach’	*kontá*	‘near’	adverb
*xak-****év****-o*	‘I hack’	*hack* (Engl.)		loanword

The ancestor of MG -*ev*-, the Ancient Greek verbalizer -*eu(-e/o-*), was originally based on *nominal* stems in -*eu*-, but could also be used to form denominal verbs from other types of nominals (column a. in Table 2). Nominal -*eu*- fell out of use on its way to Modern Greek already by the 10^th^ century (Psaltes, 1913, 254), and MG -*ev*-verbs are thus never based on nominal stems with the same suffix (column b. in Table 2; roots are underlined),^7^ unlike their AG equivalents.

**Table 2:** AG verbs in *-eúō* vs. MG verbs in *-ev-o*

a. AG verb in -*euō*		b. MG verb in *-ev-o*	
Verb	Base	Verb	Base
*basil-****eú****-ō*	*basil-****eú****-s*	*vasil-****év****-o*	*vasil-iás*_N_
‘am king; rule’	‘king’	‘govern’	‘king’
*hipp-****eú****-ō*	*hipp-****eú****-s*	*ipp-****év****-o*	*ipp-éas*_N_
‘am a horserider’	‘horserider’	‘ride’	‘horserider’
*khalk-****eú****-ō*	*khalk-****eú****-s*	*xak-****év****-o*	Engl. *hack*
‘am a coppersmith’	‘coppersmith’	‘hack’	
*arkh-****eú****-ō*	*arkhós*	*stox-****év****-o*	*stóx-os*_N_
‘command’	‘commander, leader’	‘aim at’	‘target’
*aethl-****eú****-ō*	*aethlós*	*kont-****év****-o*	*kontá*_ADV_
‘contend for a prize’	‘contest for a prize’	‘approach’	‘near’
*hēgemon-****eú****-ō*	*hēgemṓn*		
‘lead the way’	‘leader’		

AG -*eu-*verbs are denominal verbs derived primarily from agent(ive) animate nouns and nouns of profession formed with the suffix -*eu*- by adding the all-purpose verbalizer *-je/o-8 to the nominal stem, as illustrated in (12)(Fraenkel, 1906, 172–193; Debrunner, 1917, 104–108; Schwyzer, 1939, 732; Chantraine, 1948, 367–369; Risch, 1974, 332–335).

(12)**Figure:**
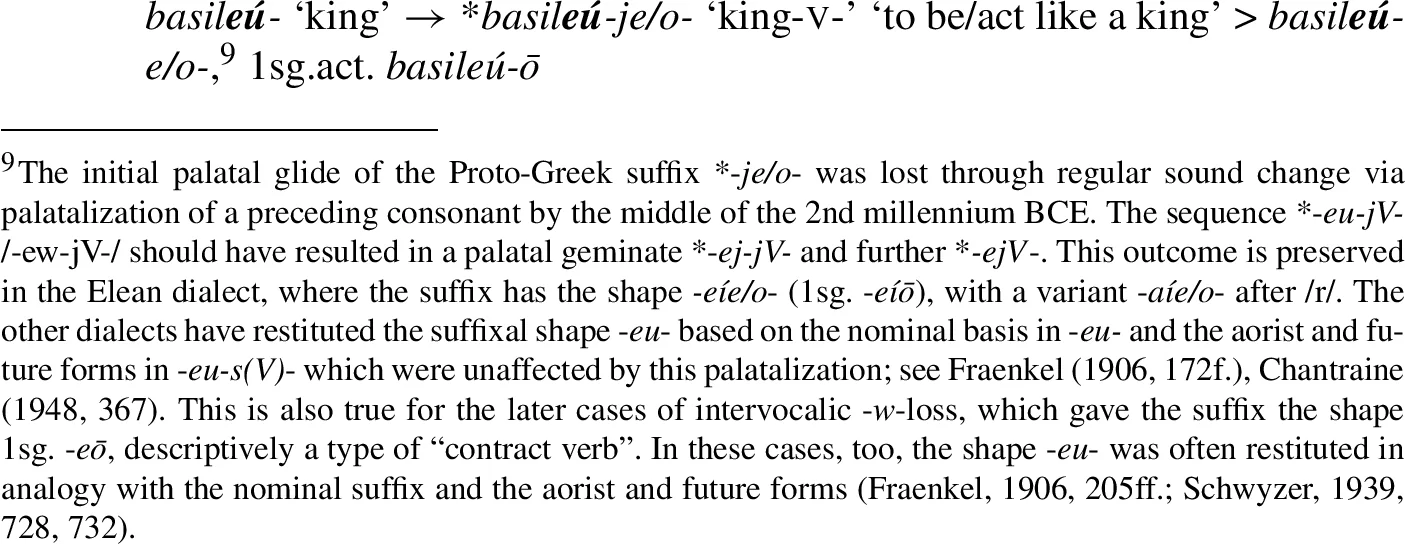

Denominal verbs in -*euō* such as (12)are thus a subclass of synchronic denominal and deadjectival verbs formed from stems that end in a consonant using the imperfective *-*je/o*- verbalizer or, in the perfective/aorist stem, the verbalizer -*s(a)*-, cf. the examples in Table 3.^10^ Semantically and in terms of productivity (specifically the ability to combine with all kinds of nominal, adjectival, and verbal bases almost without restrictions), *-*je/o*- can thus be considered a semantically bleached, default verbalizer, though we will qualify that assessment in Sect. 6.

**Table 3:**
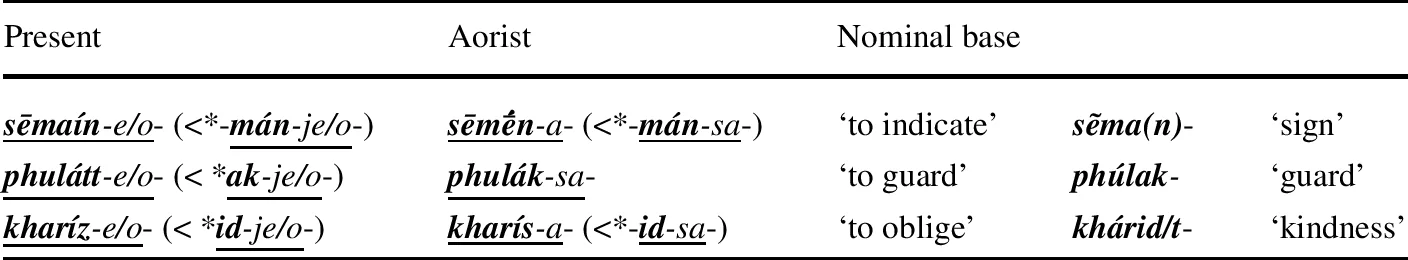
Denominal verbs from consonantal bases; nominal stem = **bold**, verbal stem = underlined

As can be gleaned from Table 3, the combination of the verbalizers *-*je/o*- and -*s(a)*- with various stem-final consonants (and vowels, in vocalic stems) led to a variety of phonotactic changes and resegmentations that gave rise to new “complex thematic suffixes”, among them precisely -*eu(-)e/o*- (see fnn. 8 and 9). Synchronically, then, the *verbal* suffix -*eu(-)e/o*- that we are interested in consists of the morpheme -*eu*- plus the theme vowel -*e/o*- to which the inflectional endings are then added. In the following, we use the first singular active ending -*euō* as the citation form as usual in the literature, except for *media tantum*/middle-only verbs, where the first singular middle -*euomai* is used. Before discussing the synchronic functions of this suffix in more detail, we first take a closer look at AG *nominal* -*eu*- in the next section.

### Nominal -*eu*- in AG

In Mycenaean and Homeric Greek, the original and dominant function of the suffix -*eu*- is the formation of animate *nouns* (Ehrlich, 1901; Bonfante, 1929; Bosshardt, 1942; Perpillou, 1973; Santiago Alvarez, 1987; Marescotti & Romagno, forthcoming). Specifically, this suffix served to derive relational nouns (‘of x’/‘in a relation with x’), where the noun in -*eu*- denotes a *y* which has some *telic quale* relation with the entity *x* denoted by the base, a definition that we adapt from Fábregas (2024, 324). In the terminology of Pustejovsky (1995), a *telic quale* “accesses the process or individual that is produced by that entity, or that the entity is designed to perform” (Fábregas, 2024, 255).

Diachronically, this suffix is usually treated as a Greek-internal innovation that arose through the combination of the inherited adjectivizer/nominalizer *-*(e)u*- with the theme vowel -*e*-11 of its nominal base (Schindler, 1976), cf. (13).

(13)**Figure:**
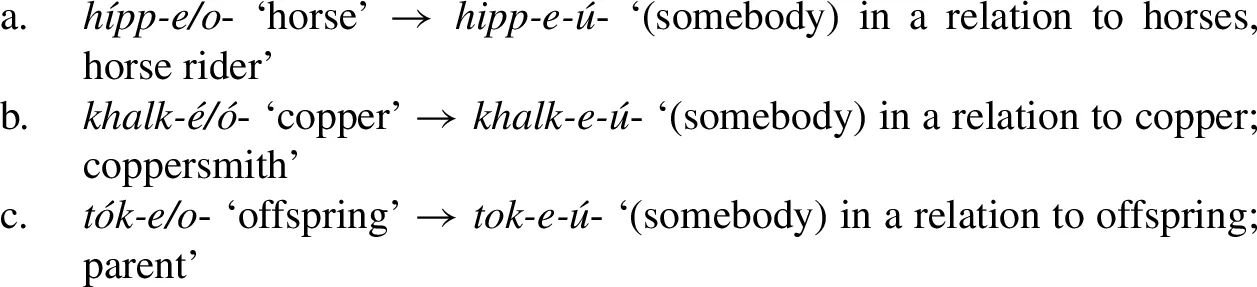

The sequence -*e-u*- was subsequently analyzed as -*eu*- and hence as descriptively replacing the stem vowel of the nominal base. Alternatively, one could consider that -*eu*- was analyzed as root-selecting at this stage, hence $\sqrt{\textsc{hipp}}$-*eu*-, etc., which would be especially attractive for cases like (13c) which can also be interpreted as semantically deverbal ($\sqrt{\textsc{tek}}$ ‘beget’, cf. 3sg.aor. *ték-e* ‘begot; gave birth to’; $\sqrt{\textsc{tok}}$-*eu*- ‘begetter; parent’). However, due to the transparently denominal meaning of the nominal -*eu*-forms in Mycenaean and Homeric Greek and their overwhelming synchronic association with *nominal* thematic bases, we think that the first segmentation with the synchronic replacement rule of the theme vowel is the better analysis.^12^ We return to the role of the nominal basis of -*eu*-nouns in Sect. 5.3.

Examples of the main functions of nominal -*eu*- in Mycenaean are given in (14)(see, e.g., Chadwick & Baumbach, 1963, 196; Perpillou, 1973, 34–45; Leukart, 1994, 240–262; Bartoněk, 2003, 279–291; Meissner, 2014; Marescotti & Romagno, forthcoming). Note that we refer to the function in (14b) as “agentive” because these nouns tend to refer to prototypical agents (that is, animate volitional entities) but are not agent nouns in the strict sense because they are not derived from verbs with an agent theta role.

(14)**Figure:**
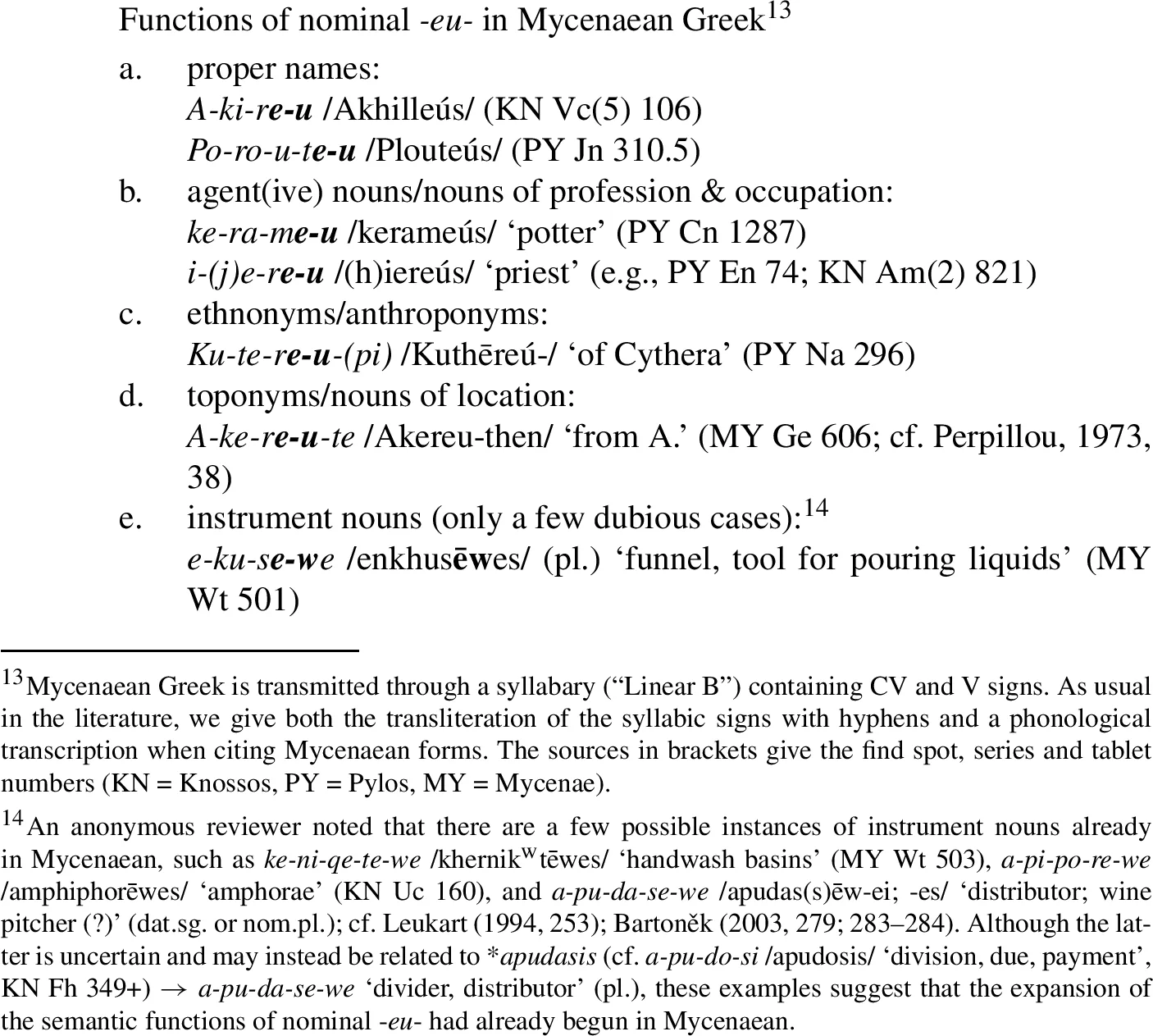

Note the similarity between these functions and the Spanish denominal suffixes denoting “jobs and occupations”, “locations”, “patronymics” and “offspring nouns” discussed by Fábregas (2024, ch. 10) as instances of the “telic quale” relation.

From the 8th c. BCE onwards, the suffix -*eu*- continues to derive proper names, (15a), agent(ive) nouns, (15b), toponyms, (15c), ethnonyms, (15d), and further expands to form instrument nouns, (15e), and nouns referring to animals, (15f) (Marescotti & Romagno, forthcoming).

(15)**Figure:**
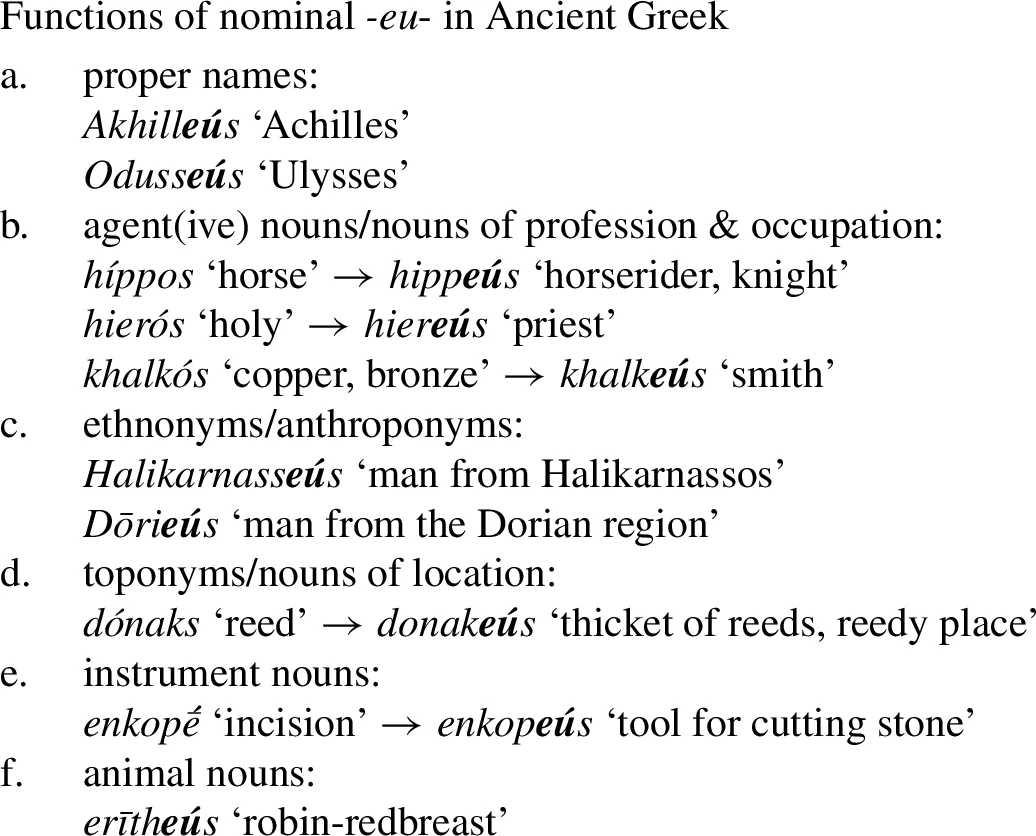

Marescotti and Romagno (forthcoming) argue that from an original core constituted by proper names, agent(ive) nouns, and ethnonyms, which share the features [+animate, +individuated], (cf. Timberlake, 1977, 162; Hopper & Thompson, 1980, 253), the functions of the nominalizer *-eu-* subsequently expanded in that 1) toponyms and nouns of location, (14d) & (15d), developed from the feature [+individuated] typical for proper names, ethnonyms, and anthroponyms; 2) instrument nouns, (15e), continue the [+agentive] feature typical of agent(ive) nouns, and 3) animal designations, (15f), developed from the [+animate] feature of proper nouns, agent(ive) nouns, and ethnonyms. Despite this semantic expansion, the most frequent function of -*eu*- across stem types is to derive agent(ive) nouns in all relevant periods (8^th^ c. BCE – 2^nd^ c. CE), as shown by Marescotti and Romagno (forthcoming), confirming that the core function of this nominalizer is to mark the animacy and the (prototypical) agentivity of its external referent. This insight is crucial to our analysis in Sect. 6, where we argue that the features [+animate], [+agentive] which characterize the functional core of the nominal suffix -*eu*- are transferred to the corresponding verbalizer in the course of the reanalysis of -*eu*- from nominal to verbal, affecting the actionality, argument structure and voice of the resulting verb. First, we turn to the syntactic and semantic properties of verbal -*eu*- in Ancient Greek in the next section.

## Verbal -*eu*- in Ancient Greek

### Data & methods

In order to determine how nominal -*eu*- became verbal -*eu*- in the history of Greek, we conducted a quantitative corpus study of AG *-euō* verbs based on the data in Fraenkel (1906), who provides a list of 280 *-euō* verbs attested from the Homeric stage (8^th^ c. BCE; for the Homeric forms cf. also Forssman, 2019) to the 1^st^ century CE. However, Fraenkel focuses on texts and inscriptions up to the period of the Attic orators (4^th^ c. BCE; Fraenkel, 1906, 2), and Koine material is not systematically discussed, especially with regard to newly formed verbs. Although the present study does not provide a systematic quantitative account of the post-Classical period, the verbalizer ultimately remained productive and survives in Modern Greek as the productive suffix -*evo* (see Sect. 3.1). Because our focus is on a change that took place at or before the earliest attested stage of Ancient Greek, we confined ourselves to Fraenkel’s earlier material, though we intend to discuss the Koine and Byzantine evidence in a follow-up study.

We gathered the occurrences of each verb using the “Full Corpus – Text Search” feature in the *Thesaurus Linguae Graecae* (TLG^15^), which allows searches of the corpus by lemma. We only included simplex forms (i.e., forms without a preverb, since preverbs can modify Aktionsart and transitivity). Following the reviewers’ suggestion, we independently validated our dataset using the morphologically annotated *Diorisis Ancient Greek Corpus* (DIORISIS^16^).^17^ We extracted all simplex *-euō* verbs from DIORISIS and subsequently isolated the Homeric attestations, which we compared with the inventory obtained from Fraenkel’s list and our TLG searches. The two procedures yielded almost identical inventories of Homeric simplex *-euō* verbs, with the exception of two verbs (*mōmeúō* and *listreúō*), which are absent from the Homeric data returned by DIORISIS. This discrepancy is most likely attributable to the different Homeric editions underlying the two corpora.

We organized the material in a database in which we recorded (1) the meaning of the verb, (2) the derivational base of the verb and its grammatical category (noun, adjective, verb), (3) the semantic class of each verb (defined below), (4) whether the verb forms agent nouns in -*tēs*/-*tēr* (see Sect. 5.3), (5) whether a cognate noun in -*eus* is attested, and (6) the first attestation of each verb by century. Since our focus is on the earliest, that is, Homeric stage, we also conducted a token analysis of the Homeric attestations of agent *-eu*- verbs and classified them according to the semantic types described below. The full list of Homeric forms and their classification is given in the Appendix. With regard to the Homeric token counts, the TLG and DIORISIS searches differ slightly: DIORISIS yields 133 Homeric occurrences of agent *-euō* verbs, whereas the TLG yields 136. This difference reflects the use of different Homeric editions in the two corpora.

For the *-eu*-verbs attested from the 7^th^ to the 4^th^ century BCE, we confined ourselves to a type analysis. In the following sections, we discuss the results of our study on the basis of these properties.

### The category of the base

Starting from Homer, verbs in *-euō* are derived from:

1. nouns in -*eu-*: *basileús* ‘king’ → *basileúō* ‘to be king, to rule’.
2. nouns formed with a stem-forming suffix other than -*eu*-: *lístron* ‘shovel’ → *listreúō* ‘to shovel, dig’, *ptōkhós* ‘beggar’ → *ptōkheúō* ‘to be a beggar, to beg’.
3. verbal stems or roots: *árdō* ‘to water’ → *ardeúō* ‘to water’; *poléō* ‘turn, plow’ → *poleúō* ‘turn, plow’. This is a small class in which the derivative usually does not differ in meaning from the base and -*eu*- acts as a synchronically productive replacement or “update” of the older verbal stem-forming morpheme.^18^ We do not discuss this class here.
4. adjectives: *lábros*, -*on* ‘furious, hasty’ → *labreúomai* ‘to talk rashly’.

Nouns in -*eu*- and other [+animate, +human] nouns represent roughly the 50% or more of the derivational bases of -*euō* verbs in each century, cf. Table 4.

**Table 4:** First attestation of -*euō* verbs per century BCE and by nominal base

Century BCE	-*eus* noun base	other [+animate] noun base	total [+animate] out of total # of -*euō* verbs
8^th^	5	18	23/ 53 (43.4%)
7^th^	3	9	12/ 25 (48%)
6^th^	11	27	48/64 (75%)
5^th^	12	33	45/94 (47.9%)
4^th^	4	22	26/37 (70.3%)

### The semantic classes of -*euō* verbs

In order to understand to what extent the nominal base of a denominal verb determines the verb’s semantic and syntactic properties, we need a descriptive framework to group our data set of AG -*euō* verbs into different semantic classes. We use a system that classifies these verbs based on the *semantic role* that the derivational base plays in the event described by the verb. For instance, in the denominal verb *hēgemoneúō* ‘to be a ruler, to command’, the nominal base *hēgemṓn* ‘ruler, commander’ plays the semantic role of an agent or the holder of a state: *hēgemoneúō* means ‘to be/act like a *hēgemṓn*’. The apparent state/event ambiguity is in fact systematic in this class of verbs and we will return to it in Sect. 5.

The semantic classification that we propose builds on the pivotal work of Clark and Clark (1979) on English denominal verbs and on its adaptation to Ancient Greek denominal verbs in Marescotti (2024, 2026). A summary of the semantic classes with an example for each class is given in Table 5. The description of each class is given below.

**Table 5:** Semantic classes of AG -*euō* verbs by derivational base

	Verb		Derivational Base		Semantic class
a.	*basil****eú****ō*	‘be king; rule’	*basil****eú****s*	‘king’	agent (/holder)
b.	*listr****eú****ō*	‘dig with a shovel’	*lístron*	‘shovel’	instrument
c.	*phut****eú****ō*	‘plant’	*phutón*	‘plant’	locatum
d.	*ard****eú****ō*	‘to water, irrigate’	*árdō*	‘water, irrigate’	activity vb
e.	*akh****eú****ō*	‘grieve’	*ákhos*	‘grief, pain’	source
f.	*orphan****eú****ō*	‘rear orphans’	*orphanós*	‘orphan’	argument
g.	*stratoped****eú****ō*	‘encamp’	*stratópedon*	‘camp’	location
h.	*hagist****eú****ō*	‘perform rites’	*hagisteía*	‘rites’	product
i.	*orth****eú****ō*	‘set right’	*orthós*	‘right’	result
j.	*nukh****eú****ō*	‘spend the night’	*núkh(t)	‘night’	time

Agent denominal verbs describe an event in which the base corresponds to the agent of the event or the holder of the state, for example, AG *basil****eú****ō* ‘to be/act like a king; to rule’ (*basiléus* ‘king’)’; Lat. *mīlitō* ‘to be/act as a *mīles*; to be a soldier; to perform military service’ (*mīles*, *mīlit*- ‘soldier’); Engl. *to* *butcher*, *to pilot*, *to nurse*, etc. These verbs are also called “pseudo-agent verbs” (Bleotu, 2019) or “behavior-related verbs” (Martin & Piñón, 2020) and they will be crucial to our argument later on in Sect. 6.

Agent verbs can also be formed from adjectives. In this case, it is the putative referent of the adjective that plays the role of an agent/holder in the derived event/state, for example, AG *nēpiakh****eú****ō* ‘to be childish, to play like a child’ (*nēpíakhos, -on* ‘childish’); AG *harpázō* ‘to be rapacious; to seize’ (*hárpaks* ‘rapacious’).

Instrument verbs describe an event in which the base corresponds to the instrument with which the action is carried out, for example, AG *listr****eú****ō* ‘to dig with a shovel’ (*lístron* ‘shovel’); AG *pharmássō* ‘to treat with drugs, to heal with medicine’ (*phármakon* ‘drug’); Lat. *asciō* ‘to cut with an axe’ (*ascia* ‘axe’); Engl. *to shovel*.

Locatum verbs describe an event in which the base corresponds to the locatum, that is, to the entity undergoing a change of location, for example, AG *phut****eú****ō* ‘to plant’ (*phutón* ‘plant’), *thōrḗssō* ‘to arm with a corslet; mid. ‘to put on a corslet’ (*thṓrax* ‘corslet’); Lat. *clipeō* ‘to put the helmet on’ (*clipeum* ‘helmet’); Engl. *to saddle*.

Activity verbs in -***eú****ō* are derived from (and often replace) morphologically simpler activity verbs, like AG *ardō* : *ard****eú****ō* ‘to water, to irrigate’. They are usually transitive with a direct object expressed in the accusative (e.g., AG *dok****eú****ō* ‘to watch narrowly’; *skal****éu****ō* ‘to stir’) and allow passivization.

Source verbs are derived from nouns that correspond to the source of the derived event or state and are thus often experiential verbs, such as AG *akh****eú****ō* ‘to grieve’ (*ákhos* ‘pain’); *tolmáō* ‘to be brave, to have courage’ (*tólma* ‘courage’); Lat. *tumultuor*, -*ō* ‘to be agitated, in confusion’ (*tumultus* ‘confusion, uproar’).

Argument verbs are derived from nouns which describe the entity the subject takes care of, that is, the patient of the event. Examples include AG *orphan****eú****ō* ‘to rear orphans’ (*orphanós* ‘orphan’) and *paid****eú****ō* ‘to teach, to take care of children’ (*paĩs*, *paid*- ‘child’).

Location verbs describe an event in which the base corresponds to the location, that is, to the target of the change of location undergone by the locatum. Examples include AG *stratoped****eú****ō* ‘to encamp, station’ (*stratópedon* ‘camp’), AG *skēnáō/skēnéō* ‘to encamp oneself’ (*skēnē* ‘tent, booth’); Lat. *(in-)retiō* ‘to catch in the net’ (*retis* ‘net’); Engl. *to* *corral*.

Product verbs describe an event in which the base corresponds to the product of the event, for example, AG *hagist****eú****ō* ‘to perform rites’ (*hagisteía* ‘rites’^19^), *psakázō* ‘to rain in small drops’ (*psakás* ‘drop of rain’); Lat. *bellō* ‘to make war’ (*bellum* ‘war’); Engl. *to flower*. This class also includes verbs derived from action nouns, such as *mátos* ‘search’ : *mat****eú****ō* ‘to do a search; to search, to seek’. These correspond to the denominal unergative activities of Hale & Keyser/Harley discussed in Sect. 2.2 that can be paraphrased as ‘to do X’. The difference between these two cases depends on the degree of concreteness and boundedness of the root, thus *hagisteía* ‘rites’ is concrete and [+bounded], while *mátos* ‘search’ is abstract and [-bounded].

Result verbs describe an event in which the base corresponds to the final result of the action: AG *orth****eú****ō* ‘to set straight’ (*orthós* ‘straight, right’); AG *helíssō* ‘to form a whirl’ (*héliks* ‘whirl’); Lat. *vastō* ‘to empty out’ (*vastus* ‘empty’); Engl. *to pulp* ‘to make into pulp’. Product and result verbs differ in that the latter are accomplishment verbs, and their lexical decomposition involves a change of state: [ (X cause) [ Y become Z ]]. Product verbs are activity verbs whose lexical decomposition involves only the operator do: [ X do ].

Time verbs describe an event in which the base corresponds to the period of time during which the event occurs, for example, AG *nukh****eú****ō* ‘to spend the night’ (*núkh(t)- ‘night’^20^); AG *hebáō* ‘to spend one’s youth’ (*hḗbē* ‘youth’); Lat. *hibernō* ‘to winter’ (*hibernum* ‘winter’); Engl. *to summer*.

Note that this classification is intentionally fine-grained, so some classes (e.g., argument and time) are expected to be relatively small because they are based on a highly specific set of lexical items. This does not preclude grouping some of these classes into broader, syntactically defined sets, of course. In the following, we moreover focus on the class that is dominant across all periods, namely the agent class. Table 4 already showed that the main derivational bases of -*euō* verbs are [+animate] nouns, among them the nouns in -*eus*. Table 6 now summarizes the overall distribution of the different semantic classes by century, that is, the number of verbs first attested in each century.

**Table 6:** Number of -*euō* verbs with respect to the semantic role of their bases per century BCE (by first attestation)

Semantic class	# of verbs per century BCE				
	8^th^	7^th^	6^th^	5^th^	4^th^
agent	**23**	**12**	**38**	**45**	**26**
instrument	4	1	4	16	3
locatum	2	1	0	3	1
activity vb	1	2	1	1	1
source	1	1	0	1	0
argument	0	3	6	8	0
location	2	1	1	7	0
product	9	2	10	5	3
result	1	1	3	5	2
time	0	0	0	2	1
unknown etymology	2	1	1	1	1
ambiguous^a^	8	0	0	0	0
Total	53	25	64	94	38

^a^For verbs that could be interpreted as either of two semantic categories, for example, agent or product. See the Appendix for examples.

Despite the semantic variation between these classes, it should be stressed that all classes share the property of requiring a [+animate] subject, in line with the core meaning of the nominal suffix -*eu*-.

The subjects of all classes, moreover, exhibit proto-agent entailments in the sense of Dowty (1979, 1991), although to different degrees. With the exception of time and source verbs, all classes instantiate subjects exhibiting a larger number of proto-agent entailments, whereas the subjects of time and source verbs exhibit fewer proto-agent entailments (cf. Dowty, 1979, 1991), where a *proto-agent* is defined as having the entailments in (16).

(16)**Figure:**
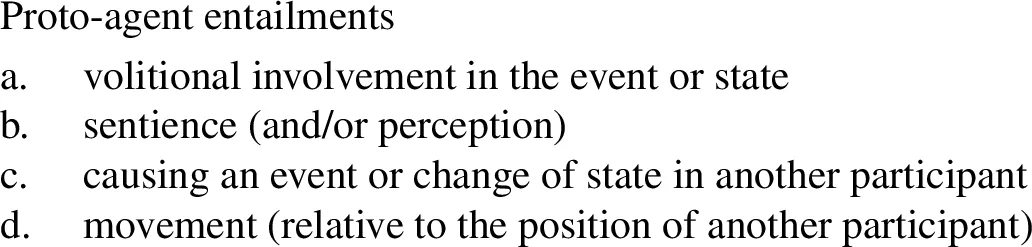

The subject of a time verb such as AG *nukheúō* ‘to spend the night’ exhibits the proto-agent entailments of volitional involvement, (16a), and sentience, (16b), but is low in causation, (16c), and movement, (16d). Likewise, the subjects of source verbs such as *akheúō* ‘to grieve’ are typically experiencers (cf. Fillmore, 1968; Dowty, 1979; Van Valin & LaPolla, 1997, 85ff.). They therefore exhibit sentience, (16b), but are typically low in volitional involvement, (16b), causation, (16c), and movement, (16d). Compared with the subjects of canonical agent verbs, the subjects of time and source verbs instantiate less prototypical proto-agents.

A remark is in order for the subject of agent verbs like AG *basileúō* ‘to be king, to rule’, *nēpiakheúō* ‘to be childish, to play like a child’: as mentioned before, agent verbs describe eventualities where the noun base can be seen as either the actor/agent of the event, (17a), or the holder of a state, (17b) (cf. Kratzer, 1996, 123).

(17)**Figure:**
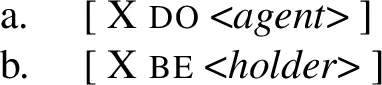

This means that, depending on the lexical decomposition given to an agent verb (i.e., state vs. activity), the logical subject may be associated with two different thematic roles (cf. Van Valin & LaPolla, 1997). Crucially, in the case of agent verbs the agentive noun base and the subject of the derived verb are co-referential, (18).

(18)**Figure:**
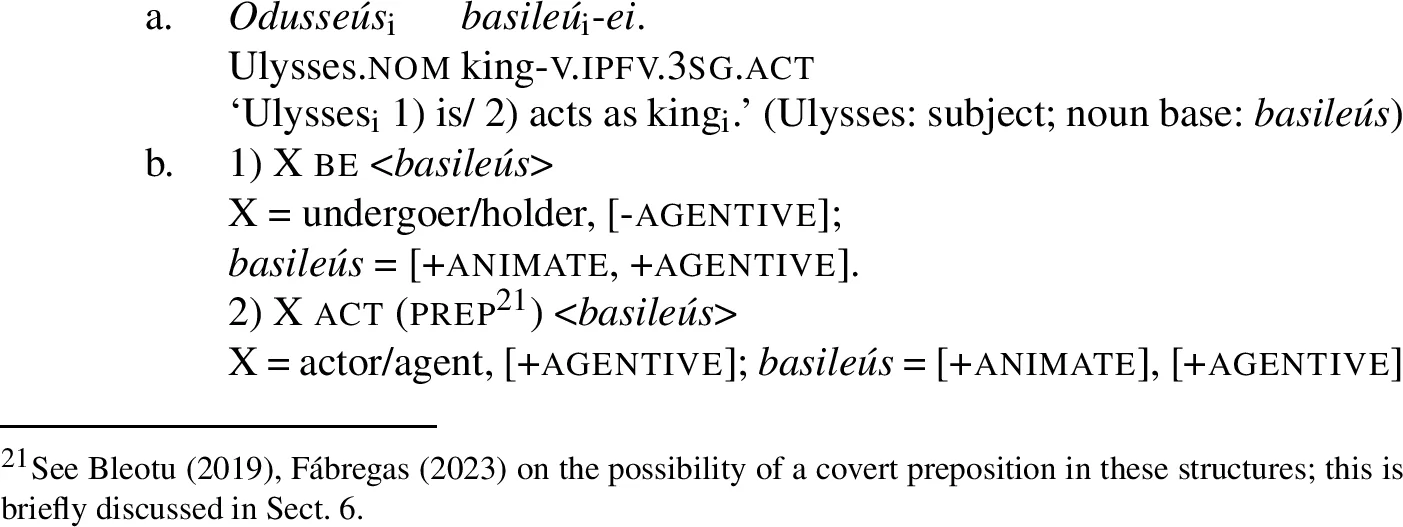

Note that co-referentiality does not require *identity* between the subject and the noun base. (19)shows that *basileúō* ‘to be/act as king’ can be said of a woman (the mother of Andromache and queen of Cilician Thebes).^22^ This suggests that the base noun is interpreted as a stage level predicate in the derived denominal verb, an intuition on which we expand in the following.

(19)**Figure:**
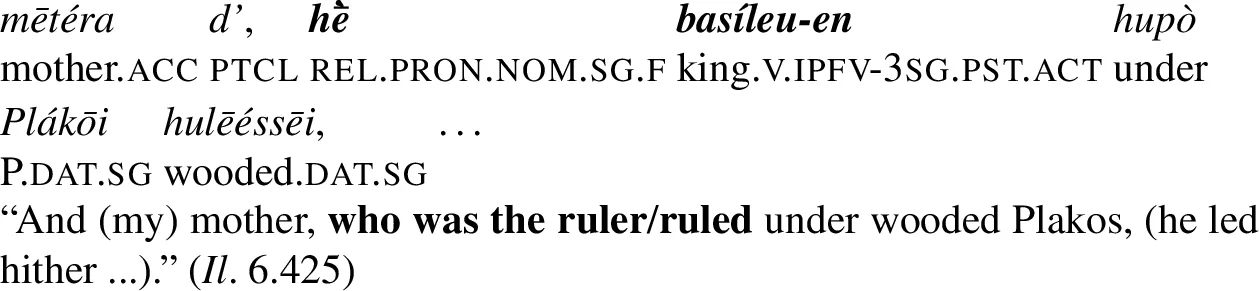

Note that in the stative reading 1) in (18b), there is a contrast between the prototypical agentivity and animacy of the noun base and the lack of agentivity of the subject of the stative agent verb, which has the thematic role of an undergoer or holder. In the activity reading 2), the thematic role of the subject aligns more closely with the semantic features of the nominal base with regard to agentivity. The question now is whether this putative semantic difference is also syntactically represented and affects the grammatical behavior of these verbs, for example with respect to their inflection. As in Modern Greek, the distribution of the Ancient Greek active vs. nonactive inflectional endings is sensitive to the thematic role of the surface subject: subjects that are merged below the canonical external argument-introducing head Voice trigger nonactive morphology, while canonical external arguments (including, and maybe limited to, prototypical agents) trigger active agreement morphology on the verb (we discuss this in more detail in Sect. 6). From this distribution it follows that denominal agent verbs should take active morphology in the activity reading and nonactive morphology in the stative reading, provided these two readings really do correspond to two syntactically different structures. We do have evidence for such alternations from denominal agent verbs without *-eu-*, for example, *anássō* ‘to be lord, to rule over’ (< *wanak(t)-je/o-; *ának-s*, *ánakt*- ‘lord’), which alternates between active and nonactive (middle) voice morphology, as in the examples in (20)–(21)(cf. Marescotti, 2024, 2026).

(20)**Figure:**
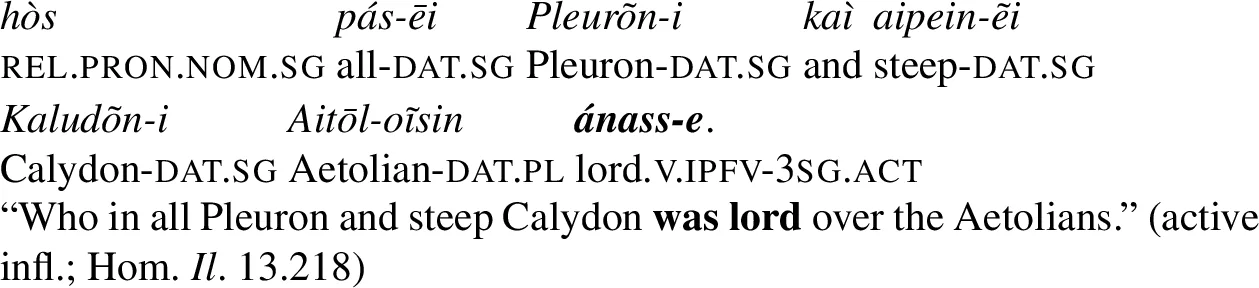

(21)**Figure:**
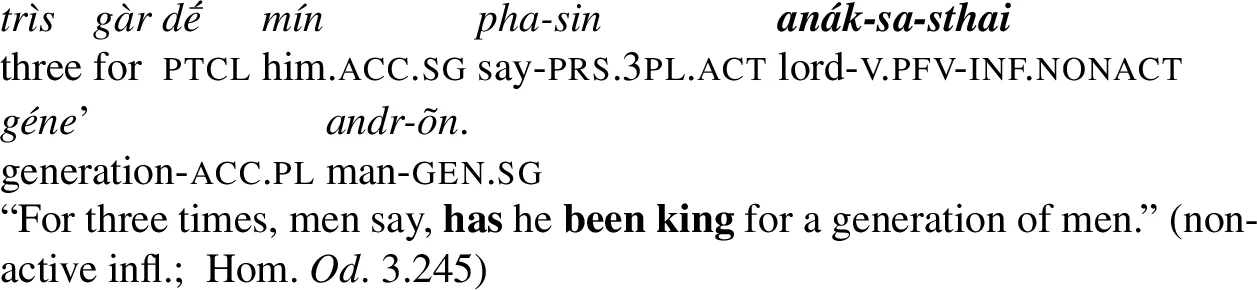

However, this alternation is usually described in the literature as occurring without any change in verb meaning, and the role of diachronic factors (such as the reanalysis of an experiential verb as an agentive verb) and synchronic speaker variation in explaining this phenomenon has not been sufficiently explored yet. We therefore set this issue aside for now, especially since there is no such variation among the Homeric agent -*euō* verbs, which are consistently active.^23^ We discuss the role of voice morphology in more detail in Sect. 6.

In the following sections, we will instead focus on the *semantic* side of this peculiar hybrid property of agent -*euō* verbs. The ambiguity between a stative and an eventive (activity) reading has been noted in the literature (e.g., Fraenkel, 1906, 177–185; Debrunner, 1917, 104–106; Schwyzer, 1939, 732), but has not received a satisfactory explanation or been linked to the rise of -*eu*- as a verbalizer. Here, we argue that it is actually crucial to understanding the diachronic reanalysis that gave rise to the categorial change of this suffix. We propose that agent -*euō* verbs originally expressed *Davidsonian states* (named after Davidson, 1967) with a [+agentive] subject and an event variable. The combination of these two properties paved the way for the reanalysis of Davidsonian states as intransitive activity verbs and hence to a change in their lexical aspect: the specific semantic features of the nominal suffix -*eu*-, namely [+animate, +agentive], were reanalyzed as belonging to the verbalizer. That is, they were effectively transferred from the surface *subject* of the denominal verb, co-referential with the agentive noun base, to the verbal event itself. Specifically, the steps we propose for this reanalysis are given in (22).

(22)**Figure:**
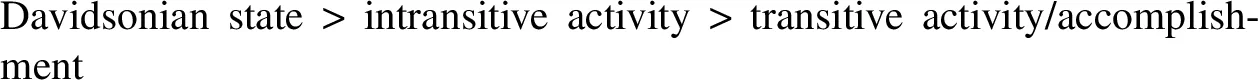

In the following, we elaborate on this analysis by focusing on the properties of the 53 -*euō* verbs attested in the 8^th^ century BCE. Given that -*euō* is already used as a verbal derivational suffix at the Homeric stage, as shown by the examples in which the nominal base does not contain -*eu*- (cf. Tables 2 and 4), and since there are no attested instances of -*euō* verbs in Mycenaean, the 8^th^ century BCE therefore provides our earliest available evidence for the shift from nominalizer to verbalizer. We begin by clarifying our approach to states vs. events in the next section.

## AG *-euō* and the state/event ambiguity

### Davidsonian vs. Kimian states

The ambiguity between a stative and an eventive reading described in the literature is specific to *-euō* verbs derived from [+animate] (, [+agentive]) nouns, independent of whether they are based on an -*eu*- noun or a different type of agentive noun. In order to understand the hybrid behavior of this class, we therefore focused on the 23 unambiguous agent -*euō* verbs attested in the 8^th^ century (types), which are attested 136 times (tokens). The full list of all 53 8^th^ BCE -*euō* verbs, their semantic classification, and their derivational base is given in the Appendix.

In order to fully clarify the nature of the apparent state/event ambiguity, we first need to determine what types of states -*euō* verbs can express and how to distinguish them from events in a corpus language. The classical and intuitive division of predicates into states versus events (Lakoff, 1966; Dowty, 1979) is insufficient here — states are not a single class in terms of their behavior, but fall into different subclasses, some of which are more similar to events than others. Maienborn (2003, 2005, 2007, 2019) proposes to call the event-like states *Davidsonian states* (D-states), contrasting them to *Kimian states* (K-states). Davidsonian states (after Davidson, 1967) are defined as “spatiotemporal entities with functionally integrated participants” (Maienborn, 2019, 55) and a “stative event argument”, exemplified by predicates such as Engl. *sit*, *stand*, *lie*, *wait*, *gleam*. Although they are non-dynamic, they pattern with eventive verbs (e.g., activity verbs) with respect to a number of syntactic tests.

Kimian states (after Kim, 1969, 1976), on the other hand, “are abstract objects for the exemplification of a property P at a holder *x* and a time *t*” (Maienborn, 2019, 71), exemplified by predicates such as Engl. *weigh*, *be intelligent/tall*, *resemble*. Standard tests show that these verbs do not contain event semantics.

**Fig. 1:**
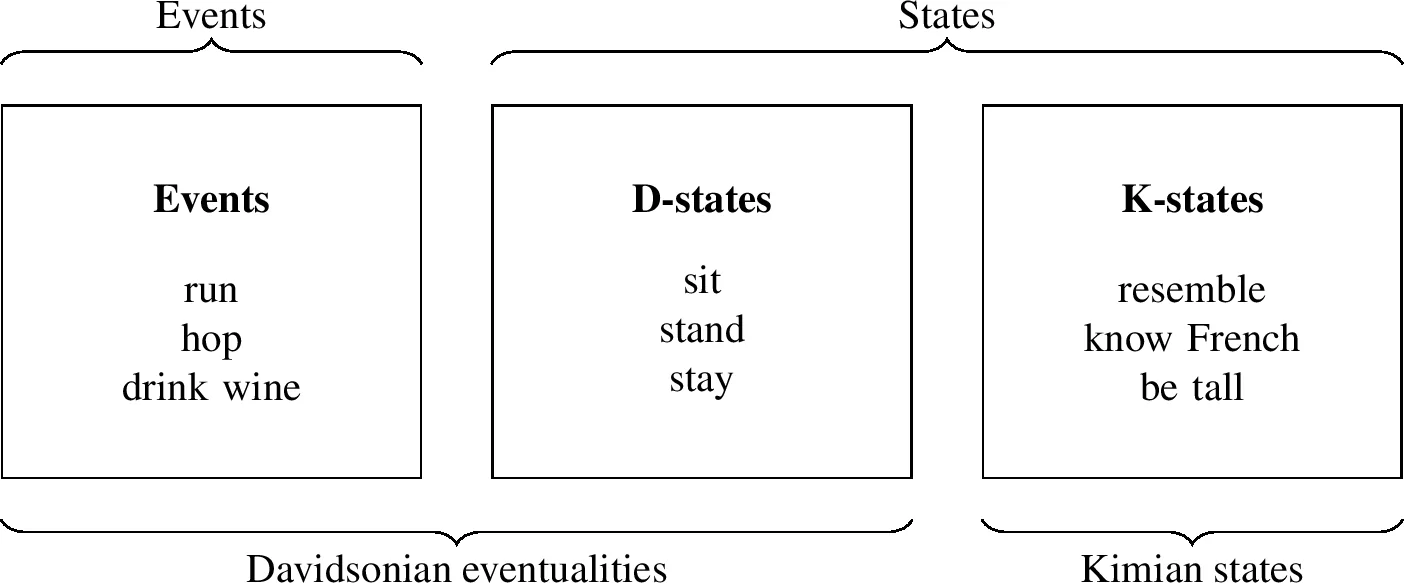
Events, Davidsonian states, Kimian states

In other words, Davidsonian states and events share the property of having an event variable, which Kimian states lack (Maienborn calls this group “Davidsonian eventualities”, which are states or events that behave as event-like individuals and can therefore be targeted by event-related modification). In contrast to Kimian states, this event variable allows them to be spatio-temporally anchored. We illustrate this overlap in Fig. 1.

Note that the distinction between D-states and K-states does not fully correspond to the distinction between stage level (SL) and individual level (IL) predication, another way of classifying states, especially among copular constructions (Carlson, 1977). The core observation in the SL/IL domain is that some predicates refer to temporal stages (*be sick, tired, angry*, etc.) and other to permanent properties of individuals (*be tall, blond, intelligent*, etc.). This distinction hinges on the question of whether the respective predicates can be located in space, the diagnostics for which are contested (see Maienborn, 2001, 2003, 2019; Ernst, 2016). Crucially, whether or not a predicate (copular or otherwise) expresses a temporary or a permanent property seems to be tangential to the question of whether or not the resulting predicate contains an event variable in its semantic structure and is accessible to event-related modification. We therefore follow Maienborn in distinguishing between predicates for which a Davidsonian event variable can be posited based on a certain set of diagnostics (on which we elaborate below) and predicates which do not contain such a variable.

The tests to discern Davidsonian from Kimian states mostly focus on detecting the event variable postulated for the former, but absent in the latter. For example, D-states are compatible with manner adverbs modifying the event, as well as event-setting locative and temporal modifiers, and they can serve as complements of perception verbs like *see*. K-states can combine with temporal modifiers, but are incompatible with manner and event-setting locative modifiers and cannot be used as complements of perception verbs (cf. Maienborn, 2007, 2019; Acedo-Matellán & Oltra-Massuet, 2024). Table 7 summarizes the relevant diagnostics discussed in Maienborn (2005, 2007, 2019) and Rothmayr (2009), rows a–e, supplemented by the diagnostics used to distinguish states from events more generally, building on Lakoff (1966), Dowty (1979), Levin and Rappaport Hovav (1995), and Levin (2009), rows f–l. Dowty already proposed that some stative verbs behave ambiguously with respect to the event vs. state diagnostics he discussed, arguably because they are D-states containing an event variable. Below, we illustrate these diagnostics with English examples before discussing their adaptation to Ancient Greek.

**Table 7:** Diagnostics for Kimian vs. Davidsonian states

		Davidsonian	Kimian
a.	manner modifiers	yes	no
b.	locative modifiers	yes	no
c.	temporal modifiers	yes	yes
d.	complement of perception vbs	yes	no
e.	second participant	yes	no
f.	progressive	yes	no
g.	imperative	yes	no
h.	compl. of force/persuade vbs	yes	no
i.	compl. of “what happened”	(yes)	no
j.	habitual reading	yes	state
k.	pseudo-cleft construction	yes	no
l.	agentivity of the subj.	±	–

To illustrate the relevant diagnostics as summarized in Table 7, compare how the K-state *own* patterns with respect to them as opposed to the D-state *sit*: while the D-state can be modified by a manner adverb (row a), (23a), the K-state is incompatible with manner modification, (23b).

(23)**Figure:**
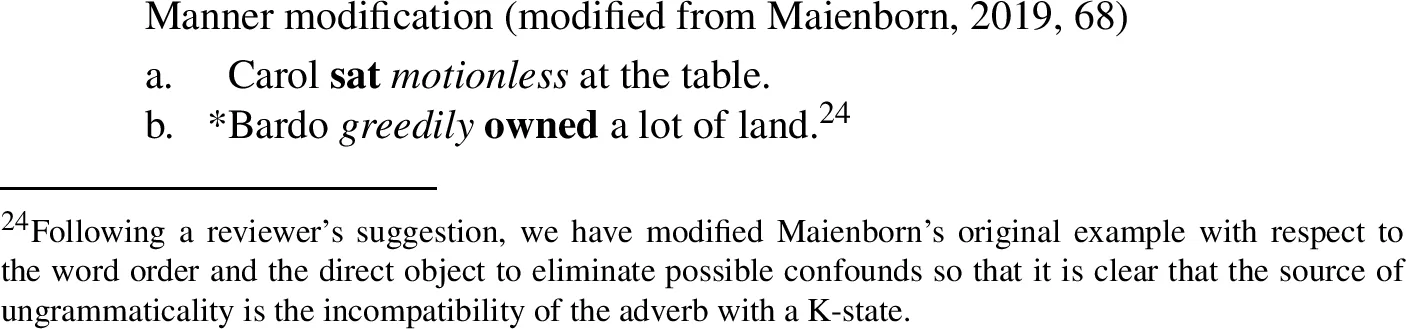

The same is the case for locative modification (row b): K-states, being abstract properties of entities, cannot be located in space, (24b), in contrast to D-states, (24a). Note that so called “frame-setting” locative modifiers are compatible both with D-states and K-states because they modify the clause/CP rather than the event; these are explicitly excluded from this diagnostic in Maienborn (2001, 2019).^24^

(24)**Figure:**
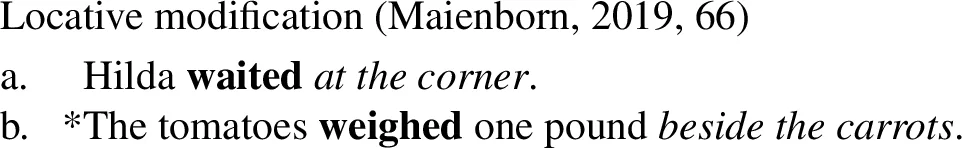

D-states and K-states also pattern together with respect to some diagnostics, for example, both D-states, (25a), and K-states, (25b), can be temporally anchored and combine with temporal modifiers (row c), (cf. Maienborn, 2007, 2019); these will therefore be excluded from the discussion.

(25)**Figure:**
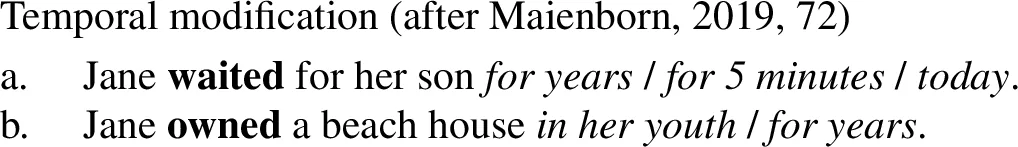

Other diagnostics are more clear-cut: Only D-states can serve as complements of perception verbs (row d), (26a). K-states, being properties of individuals, cannot be perceived, (26b).

(26)**Figure:**
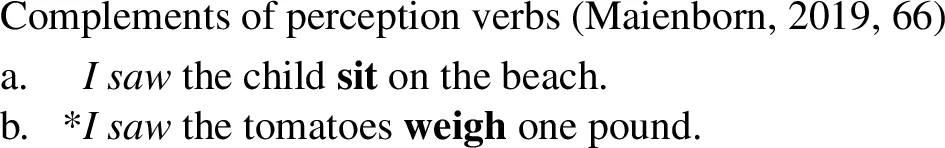

Only D-states admit the presence of additional participants in the event (row e), (27a). Since there is no event in K-states, no event participants can be added, (27b) (note that Maienborn subsumes these examples under “manner modification”).

(27)**Figure:**
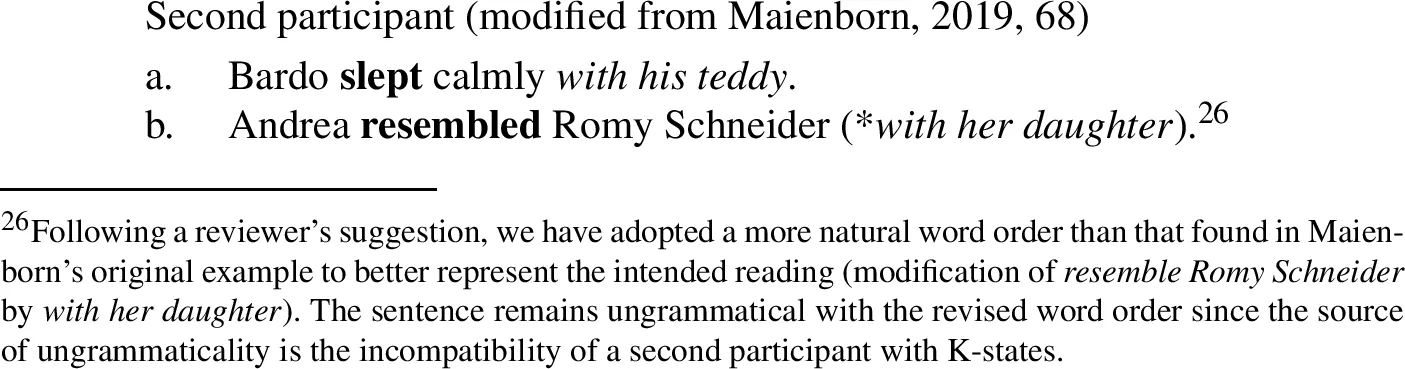

A K-state such as Engl. *know* cannot occur in the progressive construction (row f), (28b), unlike D-states such as *wait*, (28a) and dynamic events such as *run*, (28c).

(28)**Figure:**
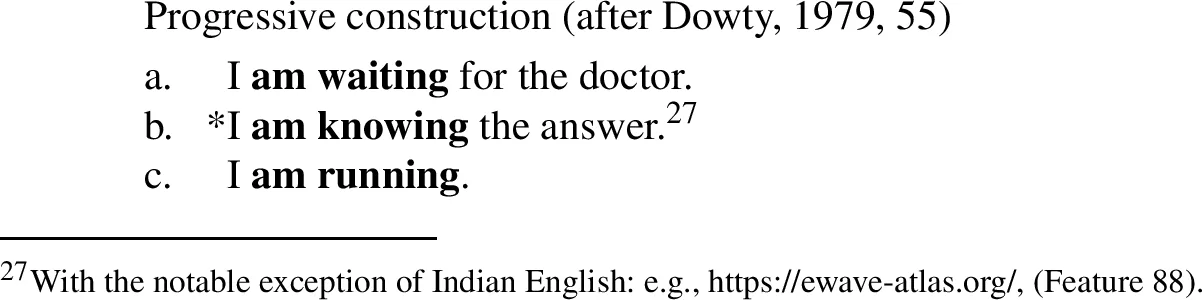

D-states and eventive verbs also pattern together with respect to their ability to occur in the imperative mood (row g), (29a,c), while K-states cannot usually occur in the imperative in out-of-the-blue contexts, (29b), though this and the previous diagnostic have been noted to be susceptible to coercion of event readings.^27^

(29)**Figure:**
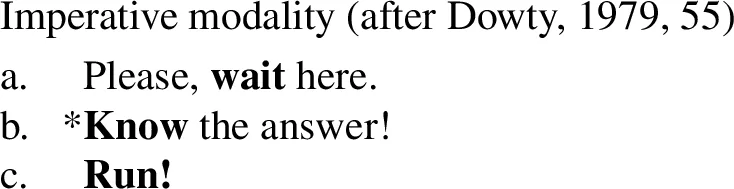

Only D-states and eventive verbs can serve as a complement of verbs of force and persuasion (row h), (30a,c); K-states cannot, (30b) (after Dowty, 1979, 55; cf. Levin, 2009).

(30)**Figure:**
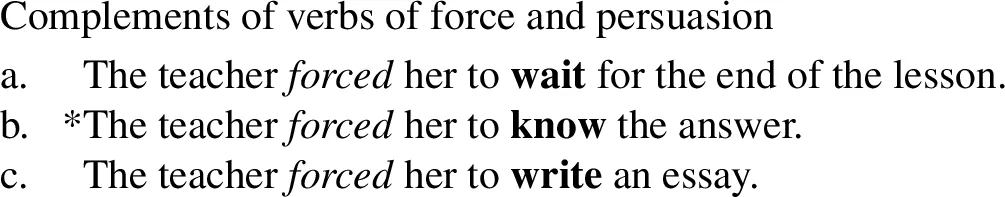

Only D-states and event verbs can serve as a complement of a “what happened” verb (row i), (31a,c); K-states cannot, (31b).

(31)**Figure:**
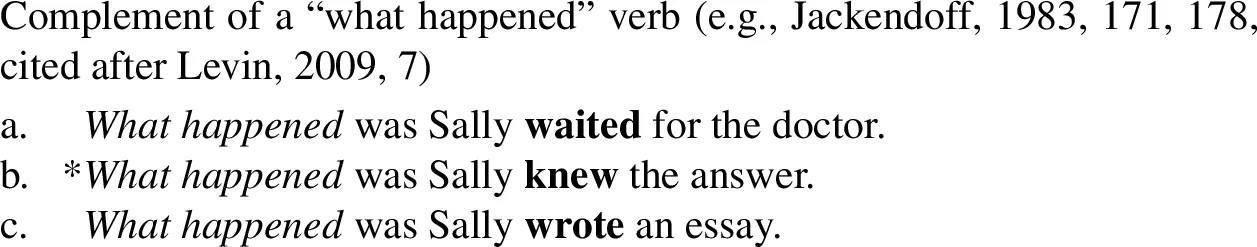

However, some “posture verbs” such as *sit*, *stand*, and *lie*, which otherwise pattern as D-states according to Maienborn’s diagnostics, behave like K-states with respect to the “what happened was” test and the pseudo-cleft test (row k) in lacking agency and change-of-state readings (see Gómez-Vázquez & Mateu, 2022 for discussion), while verbs like *know* can be coerced into an inchoative reading in contexts like (31b) (cf. *Suddenly, I knew the answer*, De Swart, 1998, 359), so these diagnostics also need to be carefully negotiated when adapting them to a corpus language.

In English, the use of the simple present with eventive verbs and D-states results in a habitual reading (row j), as in (32a,c), whereas the episodic reading is expressed with the present progressive (32b,d). K-states, instead, do not distinguish the habitual from the episodic reading, since they express a property of the subject and receive a habitual reading by default. Hence they also cannot occur in the progressive, cf. (32f) and (28).

(32)**Figure:**
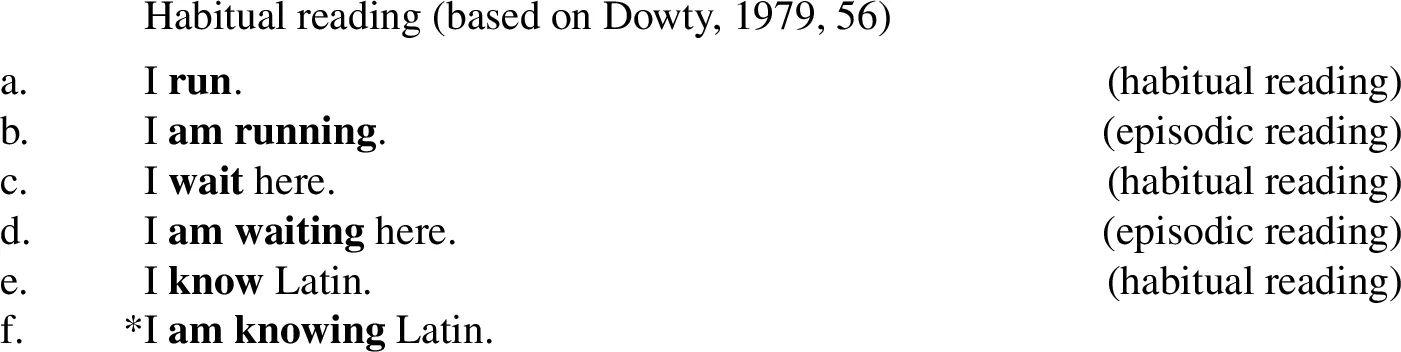

Only D-states and eventive verbs can occur in pseudo-cleft constructions (row k), (33a,c), unlike K-states, (33b).

(33)**Figure:**
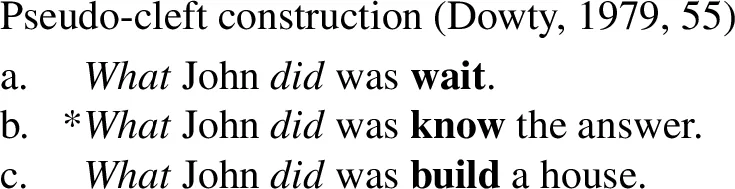

Finally, eventive verbs and D-states can have an agentive subject or a subject which shares some properties with a prototypical agent (row l), while the subject of a K-state is never agentive in the sense of Dowty’s “proto-agents” (Dowty, 1979, 1991), cf. (16).

In the following section, we discuss how these sets of diagnostics can be adapted and applied to our Ancient Greek data.

### Davidsonian vs. Kimian states in Ancient Greek: adapting the diagnostics

In the previous section, we discussed different tests proposed in the literature for detecting Davidsonian states, that is, states containing an event variable. Most of these diagnostics come from work on contemporary Indo-European languages, in particular English, German, and Spanish, and they differ from language to language (for example, depending on whether or not a language has a designated progressive construction, that diagnostic may or may not be used). We now need to adapt these diagnostics to Ancient Greek to determine whether AG agent -*euō* verbs express K-states, D-states, or both. The basic idea behind this is that K-states essentially lack any sort of event structure, so if AG denominal agent verbs in -*euō* were originally K-states, it would be difficult to explain how they acquired an event variable and became activity verbs (or other types of eventive verbs). If we can show that agent -*euō* verbs pattern with D-states with respect to the relevant diagnostics, on the other hand, this would be evidence that they contained an event variable from the very beginning, which could then have formed the basis for further development of -*eu*- into an eventive verbalizer. Because we are dealing with a corpus language, we have to rely on positive evidence only, and evidence for a D-state reading means that this was at least possible at this stage of the language, but does not as such exclude the possibility of an (unattested) K-state reading. We return to this point below in the discussion of the findings.

Our first task was to find AG equivalents to the diagnostics discussed in the previous section. Table 8 summarizes these diagnostics and their proposed AG equivalents. We found equivalents for almost all diagnostics (as mentioned in the previous section, we excluded the temporal modifier diagnostic), except for the progressive construction, since AG does not have progressive aspect. Ionic Greek, one of the main dialects underlying Homeric Greek, does have a construction usually referred to as “iterative preterit”, which is formed by adding the “iterative” suffix -*ske/o*- to the imperfective (present) or perfective (aorist) stem of the verb. The exact semantics of this construction are contested, but it is usually described as expressing iterative, durative, and/or frequentative aspect, depending on whether it combines with the imperfective or the perfective stem (see Schwyzer, 1939, 710f.; Chantraine, 1984, 226f; Ittzés, 2008; Kimball, 2014 for discussion). Based on this assessment and the combinatorial properties of the iterative suffix, Grestenberger (2022) argues that it expresses a type of imperfective viewpoint aspect with iterative or frequentative flavor. This is not the same as progressive aspect, but closer to what is usually translated with the auxiliary *keep* in English, which is again compatible with D-states (*Livia keeps waiting in the doctor’s office*) but not with K-states (*Livia keeps knowing the answer, *keeps being intelligent). Since K-states are incompatible both with the progressive and with iterative/frequentative aspect, we therefore use the Ionic iterative preterit as a stand-in for the progressive and habitual/iterative diagnostics.

**Table 8:**
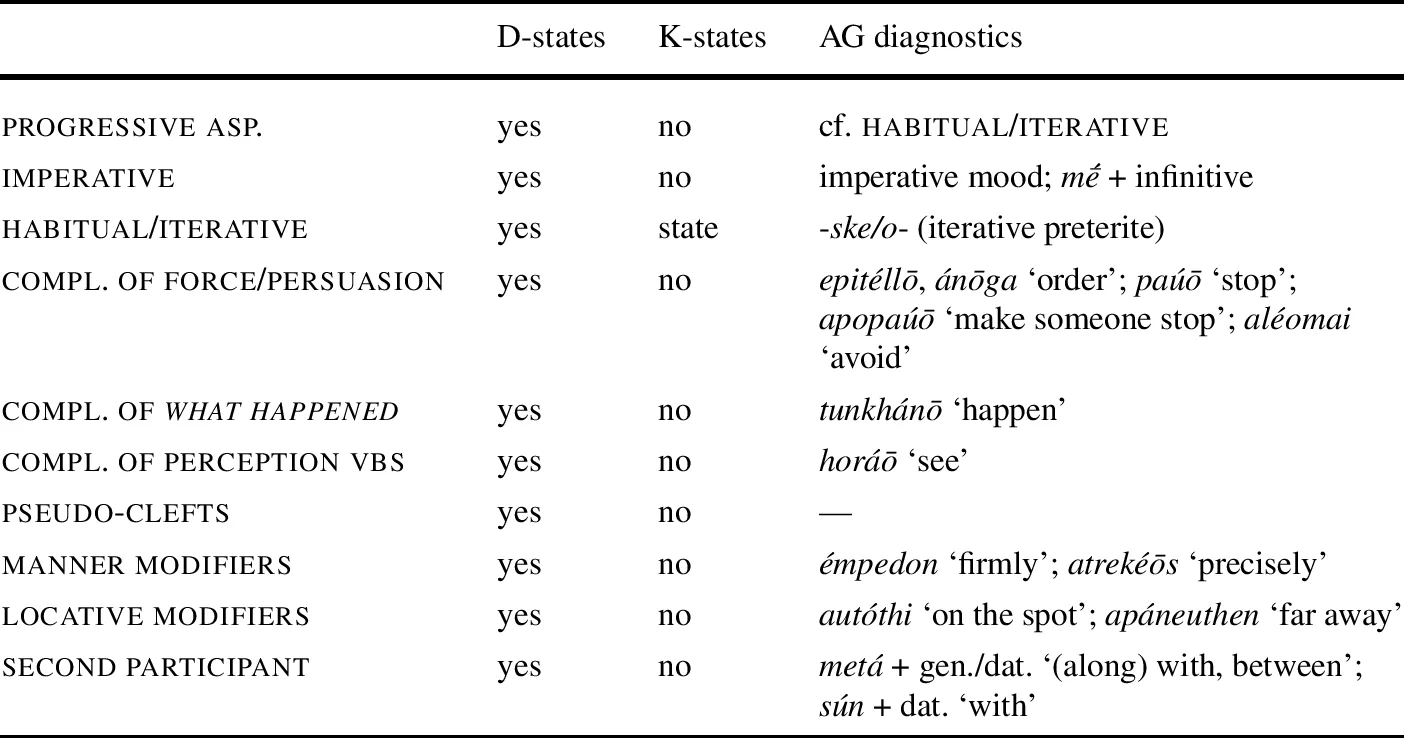
Diagnostics for D- vs. K-states and their adaptation to AG

We also left aside the pseudo-cleft construction diagnostics, since a study of the properties of Ancient Greek pseudo-cleft constructions — especially *wh*-clefts — or their information structural equivalents is beyond the scope of this paper (see Banti, 2013; Probert, 2015 for surveys of comparable cleft and relative clause constructions). A more detailed discussion of the remaining diagnostics with examples follows below.

The first relevant diagnostic, compatibility with imperatives, is illustrated in (34)for the agent verb *mant****eú****omai* ‘to be/act as a prophet, prophesy’, derived from the [+animate, +agentive] noun *mantís* ‘prophet’, or possibly unattested *manteús ‘prophet’. AG uses designated inflectional imperative endings on the verb to express imperative mood, as in (34), as well as a construction consisting of *mḗ* ‘not’ and the infinitive to express negative imperatives.

(34)**Figure:**
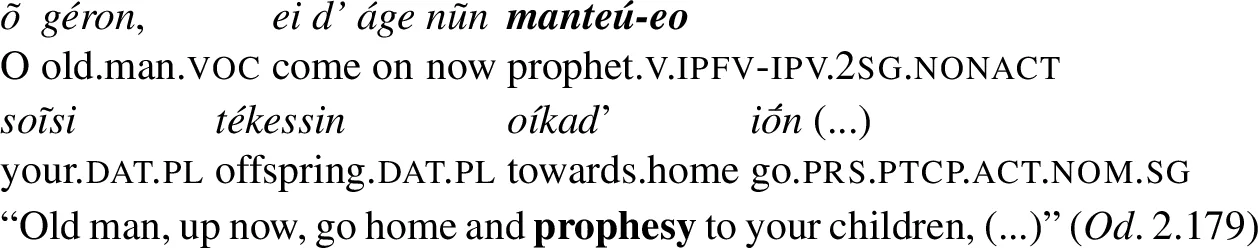

Compatibility with iterative or habitual aspect is illustrated in (35), in which the iterative preterit suffix -*ske/o*- combines with *aristeúō* ‘to be the best’ (from *áristos* ‘best’ or *aristeús* ‘best man, chief’).

(35)**Figure:**
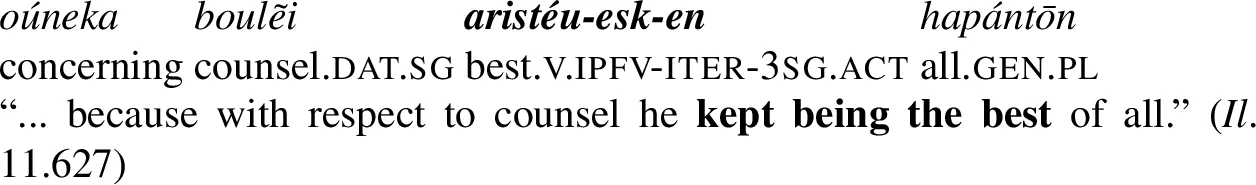

Agent -*euō* verbs can occur as complements of verbs of force and persuasion such as *epitéllō* ‘order, impose’, (36a), *ánōga* ‘order, bid’, (36b), or *paúō, -omai* ‘bring to an end, stop, cease’, (36c). In (36a), *aristeúō* ‘be the best’ is used in the complement, in (36b) it is *pontoporeúō* ‘be a sea-farer, sea-fare’ (from *pontopóros* ‘sea-faring, sea-farer’^28^) and in (36c) it is the deadjectival verb *nēpiakheúō* ‘be childish; play like a child’, derived from the [+animate] adjective *nēpíakhos, -on* ‘childish’.

(36)**Figure:**
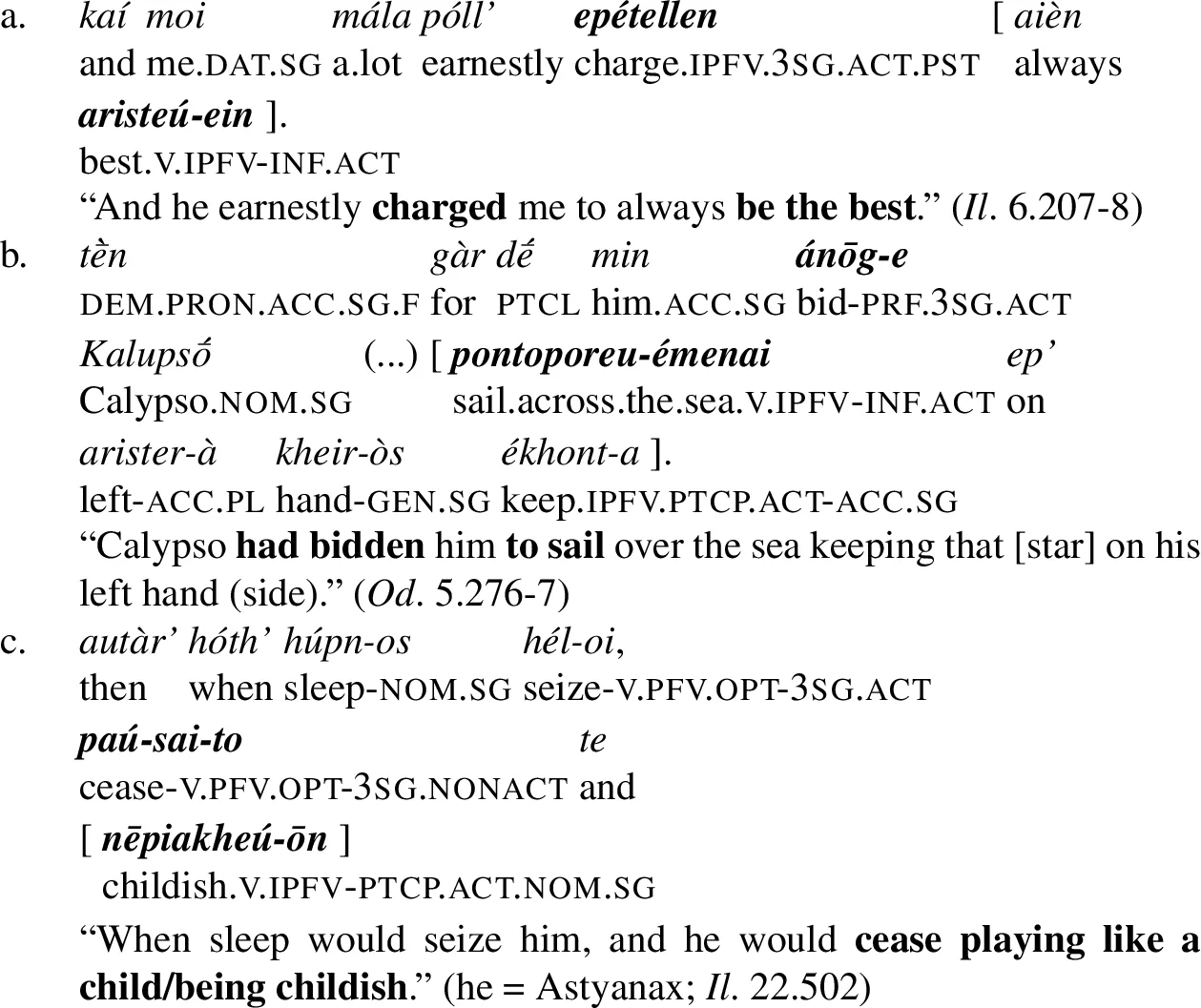

Modification by manner modifiers is illustrated in (37), in which the verb *manteúomai* is combined with the manner adverb *atrekéōs* ‘exactly, accurately’.

(37)**Figure:**
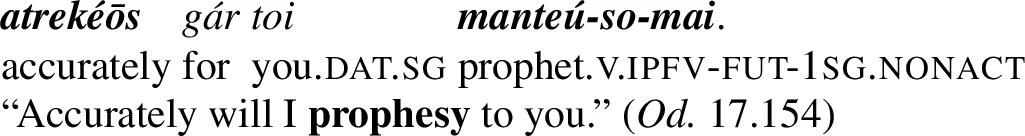

An additional example of a agent -*euō* verb combined with manner modifiers is given in (38), in which the verb *hēniokh****eú****ō* ‘to be/act as a charioteer; to drive’ (from *hēniokh****eú****s* ‘charioteer’) is modified by the manner adverb *émpedon* ‘firmly, steadily’.

(38)**Figure:**
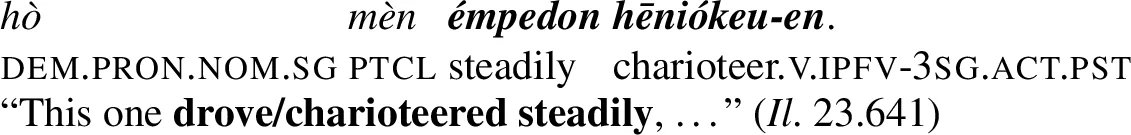

Concerning locative modification, as discussed in Sect. 5.1, Maienborn (2019, 37, 41-43) distinguishes between event-setting and frame-setting locatives, as illustrated in (39).

(39)**Figure:**
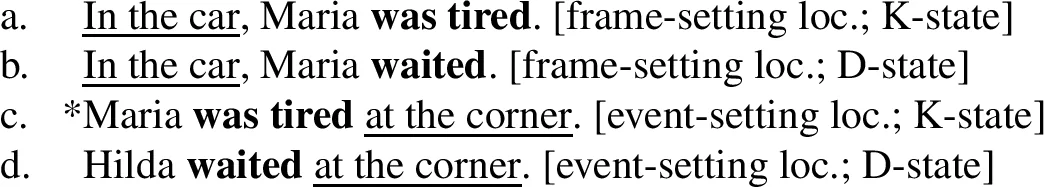

Frame-setting locatives often yield temporal or conditional interpretations (for example, ‘When/if she is in the car, Maria is tired’ for (39a)) but might also be interpreted epistemically, for instance (‘According to her mother, Maria is tired in the car’).^29^ Frame-setting locatives as in (39a, b), are compatible with both Davidsonian and Kimian states, because they modify the entire clause, rather than the event.^30^ On the contrary, event-setting locatives (39c, d) cannot combine with Kimian states but only with Davidsonian ones (cf. also Acedo-Matellán & Oltra-Massuet, 2024, 5–6), because they modify the verb, and, as discussed in Sect. 5.1, only events/eventive verbs may be located in space, not K-states.

Consider the following examples, involving the verb *hēgemon****eú****-ō* ‘be/act as chief, leader; lead’, derived from the [+animate, +agentive] noun *hēgemṓn* ‘chief, leader’, (40).

(40)**Figure:**
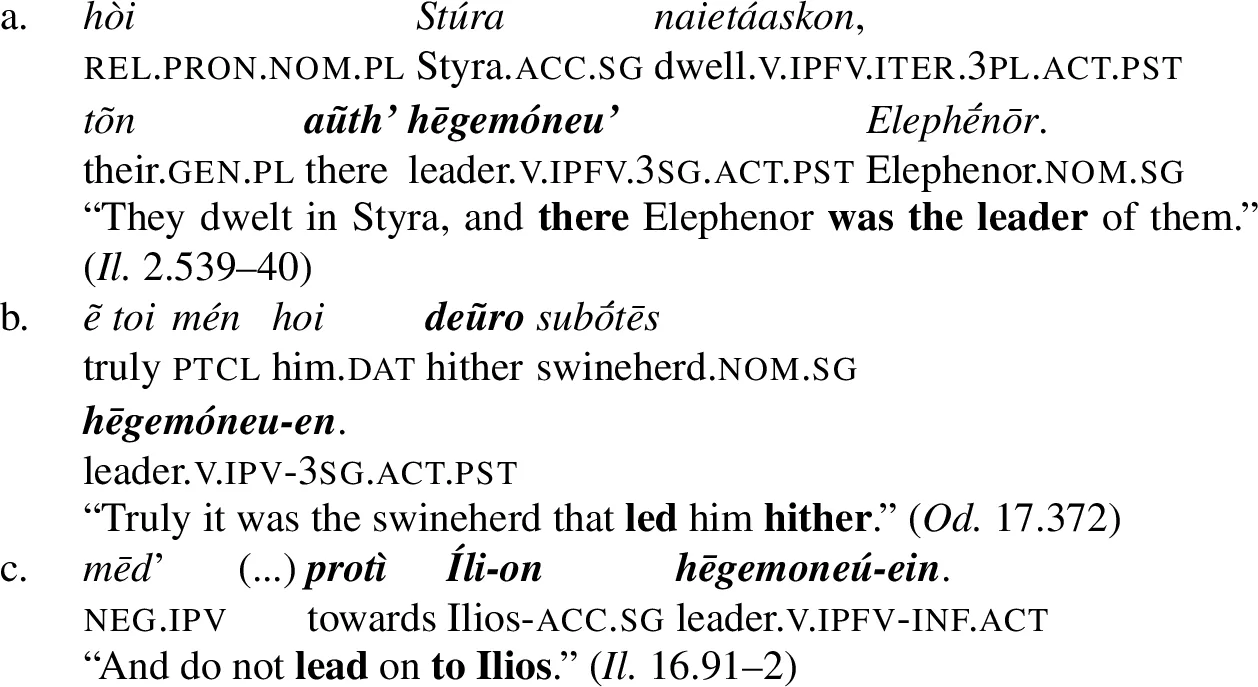

In (40a), the verb *hēgemoneúō* is combined with the locative modifier *aũthi* ‘there/here’, which at first glance may look like a frame-setting locative. However, an analysis of the occurrences of the particle from Homer on using the TLG reveals that it never occurs in copular constructions and that it is usually combined with interval state verbs like *ménō* ‘to remain’ and *hẽmai* ‘to sit’. This suggests that *aũthi* should be considered an event-setting modifier in (40a).

In (40b,c), on the other hand, the verb is combined with locative modifiers which can only be interpreted as event-modifying, namely *protì* *Íli-on* ‘towards Ilios’ and *deũro* ‘hither’, both of which are directional modifiers that usually combine with dynamic verbs of motion (cf. Cunliffe, 1924, 88, 348).

Finally, agent -*euō* verbs admit a second participant to the event, (41).

(41)**Figure:**
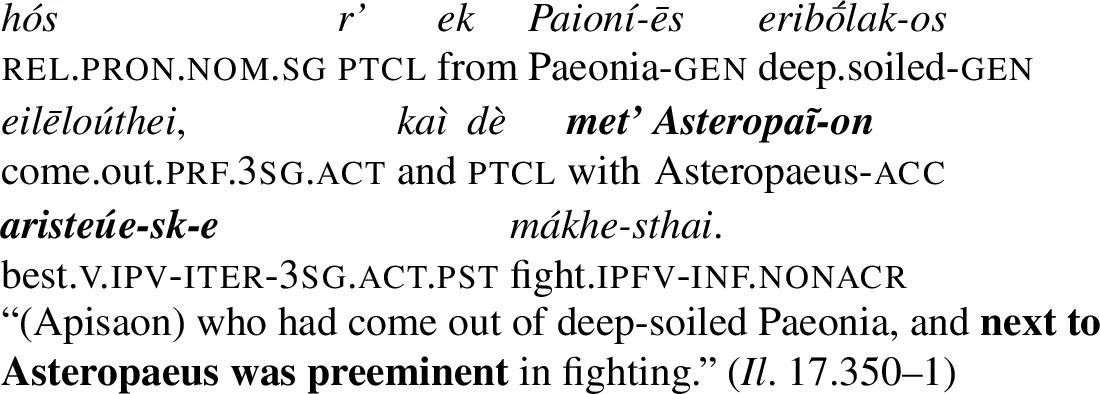

The results of our token analysis of Homeric agent -*euō* verbs are summarized in Table 9. For this part of the study, we extracted the attested instances of agent -*euō* verbs (136 out of a total of 312 attestations of 8^th^ BCE -*euō* verbs) and checked whether they appeared with one or more of the relevant diagnostics in these passages. What the results in Table 9 and the examples above clearly show is that Homeric agent -*euō* verbs are compatible with a Davidsonian state reading at this stage of the language, and we believe that this is precisely the source of the apparent state/event ambiguity noted for these verbs in the literature.

**Table 9:**
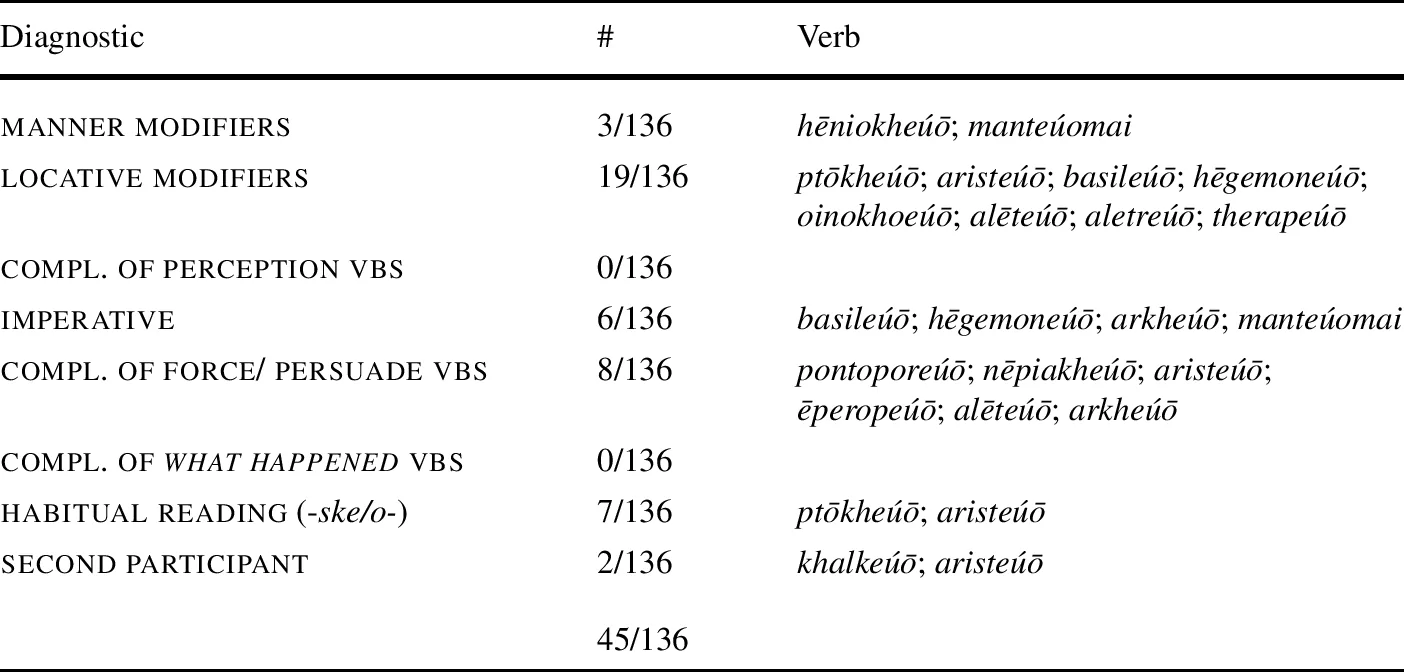
K- & D-state diagnostics applied to Homeric deagentive -*euō* verbs

As an additional test of the analysis proposed above, we conducted a random-sample study of a set of clear Kimian states, that is, non-spatially anchored properties of individuals, in Homeric Greek, assessing their behavior with respect to the diagnostics discussed above. Even though this does not prove ungrammaticality of these structures in the strict sense, it shows that among a comparable sample of tokens of presumed Davidsonian vs. Kimian states, only the former appear in the relevant event-diagnosing structures.^31^ That is, if the Kimian states under consideration consistently differ from the -*euō* verbs examined with respect to these diagnostics, this provides independent support for the validity of the diagnostics in distinguishing between Kimian and Davidsonian states.

We examined a sample of the following Kimian states: copular *eimí* ‘be’ + kinship terms (*mḗtēr* ‘mother’, *patḗr* ‘father’, *thugátēr* ‘daughter’) (15 tokens); *kaínumai* ‘to excel’ (19 tokens); *epístamai* ‘to know’ (28 tokens); *huperékhō* ‘to be superior’ (1 token); *éoika* ‘to resemble’ (151 tokens). The results show that these predicates do not occur with manner and locative modification, do not combine with the habitual suffix -*sk*-, do not occur as complements of perception, persuasion, or *what happened* verbs, and do not occur in the imperative in our sample. Furthermore, they do not license the presence of secondary participants. These findings are summarized in Table 10.

**Table 10:**
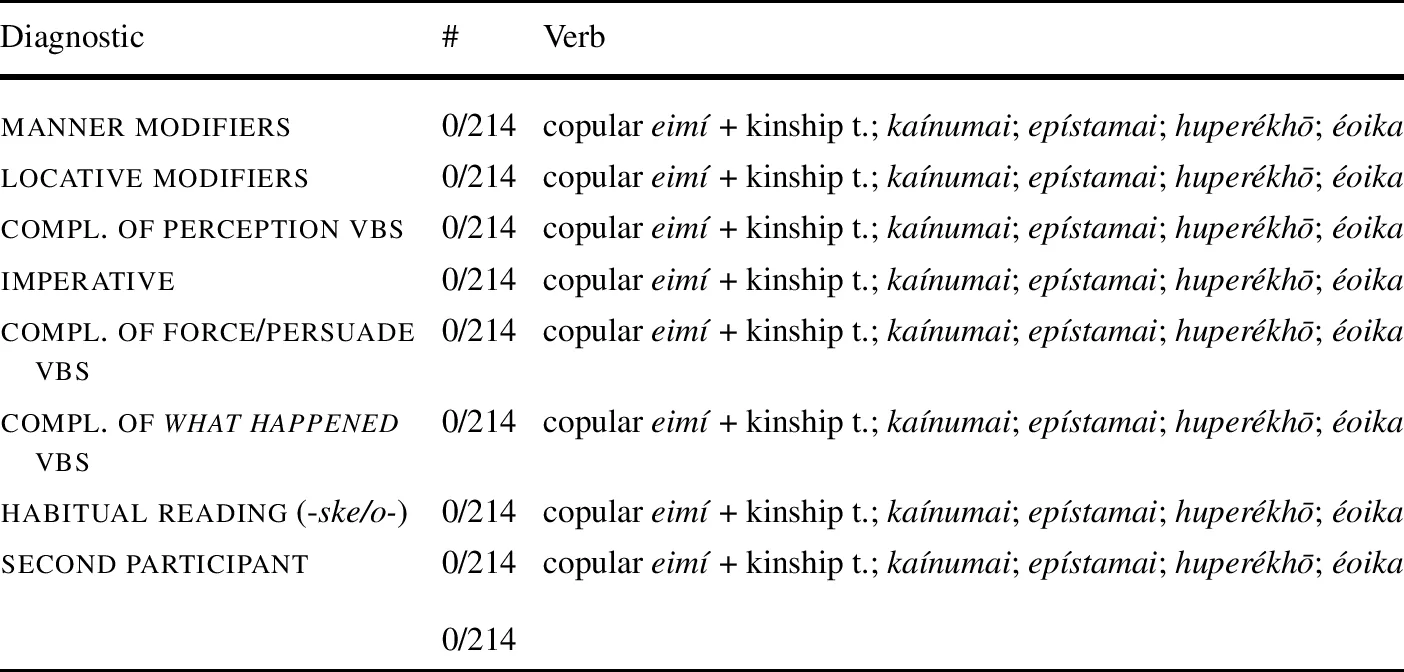
K- & D-state diagnostics applied to Homeric K-states

The contrasting distribution of Kimian states and -*euō* verbs in the corpus indicates that the diagnostics proposed above reliably distinguish between predicate types and can therefore be used to identify the presence of an event variable in Ancient Greek -*euō* verbs.

### Homeric -*euō* verbs and the path to transitivization

In the previous section, we have shown that (intransitive) agent -*euō* verbs were Davidsonian states from the earliest attested stage on. In this section, we briefly discuss their further development, and in particular, the properties of the non-deagentive -*euō* verbs attested in the 8^th^ century, many of which are clearly eventive without any trace of the state/event ambiguity associated with the agent class. We believe that in the course of the reanalysis that turned nominal -*eu*- into verbal -*eu*- (detailed in Sect. 6), the event variable already contained in D-states became associated with -*eu*- itself and further extended to new contexts and bases, on the one hand through transitivization (the acquisition of a direct object), and on the other hand via derivation from nominal bases other than Agents, but which still require a [+agentive] subject (for instance, instrument noun bases, e.g., *lístron* ‘shovel’: *listreúō* ‘to dig with a shovel’). Related to the first point, we also found a few instances of passivization, though this property is rare at the earliest stage.

Starting from the “stative event” variable in Agent -*euō* verbs, these verbs, although originally intransitives, developed into activity and accomplishment verbs and gained the ability to occur with a direct object. This process was well underway already at the Homeric stage. Of the 136 occurrences of Agent verbs, 29 involve a transitive use of the verb with an accusative object, as in the examples in (42). The verb *ptōkheúō* ‘to beg’ in (42a) is derived from *ptōkhós* ‘beggar’, so this is an Agent verb in which -*eu*- is already used as a verbalizer, while *ēperopeúō* ‘to cheat’ in (42b) is derived from *ēperopeús* ‘cheater’.^32^

(42)**Figure:**
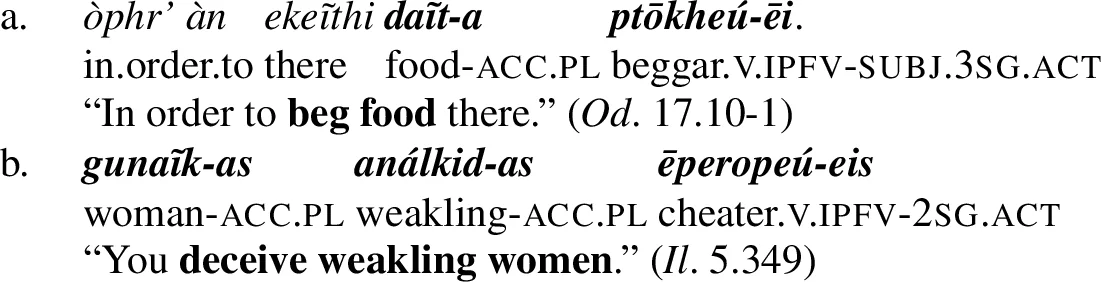

We propose that the starting point for transitivization of Agent verbs was the use of these verbs with accusative “pseudo-objects” that are closer to an experiencer or goal accusative rather than a structural internal argument. Thus the food in (42a) is the goal of the predicate *beg*, while (42b) is closer to an experiencer accusative (cf. Lowe, 2013; Lowe, 2017, 267–8 on the “accusative of experiencer”). Experiencer/goal accusatives can be subjects in several older Indo-European languages (cf. Danesi, 2014; Barðdal et al., 2020), though not in Ancient Greek, where they behave syntactically more like adverbial modifiers meaning “with respect to X” (cf. the discussion of the “accusative of respect” in Schwyzer, 1950, 84–6; van Emde Boas et al., 2019, 365–6). These accusatives would originally have differed syntactically from structural case accusatives (i.e., theme/patient accusatives), but became reanalyzed as thematic direct objects when *-eu-e/o*- became an activity verbalizer. This account explains why we find a relatively high number of transitive uses of -*euō* verbs already at the Homeric stage but a distinct lack of passivization at this stage: Experiencer/goal objects cannot become the subjects of passives at this early stage of Greek.^33^

If we include the non-deagentive -*euō* verbs as well, the Homeric examples of -*euō* verbs combined with an accusative object are 132 (out of 312) in total, suggesting that the transitivization process was well on its way already at this early stage. Examples of transitive uses of -*euō* verbs derived from product and instrument noun bases are given in (43). The verb *sūleúō* ‘to despoil, seize, rob of’ in (43a) is based on *sū́lē* or *sũlon* ‘(right of) seizure, confiscation’; *listreúō* ‘to shovel, dig’ is from *lístron* ‘shovel’.

(43)**Figure:**
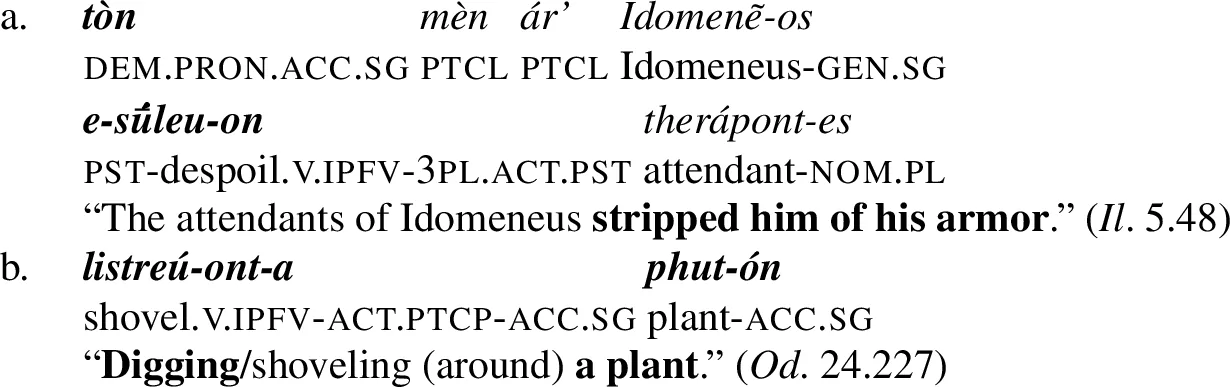

While the accusatives in (43)are still analyzable as non-canonical direct objects (such as “accusatives of respect” *vel sim*.), we also find verbs whose accusative object behaves more like a canonical internal argument, as in the case of transitive *hiereúō* ‘to sacrifice’, which always takes a direct object in Homer, see (44). Etymologically, this verb is ambiguous between a Product and an Agent verb, that is, either ‘do *hierá*’ in which -*eú*- is already a verbalizer or ‘act (as)/be *hiereús*’ from *hiereús* ‘priest; sacrificer’ with older nominal -*eu*-. Synchronically, however, it behaves as a regular transitive accomplishment verb.^34^

(44)**Figure:**
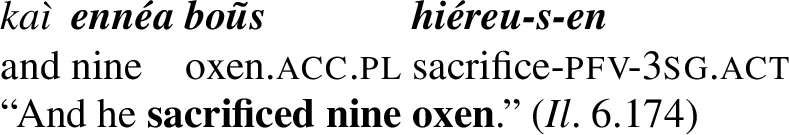

The same Agent/Product ambiguity may also have been at play in the transitivization of, for example, *khalkeúō*, *nomeúō*, *pompeúō*, which could have become reanalyzed as derived from the respective bases *khalkós* ‘copper’, *nomós* ‘pasture’, *pompós*/*pompḗ* ‘conduct, escort’ of the attested nouns in -*eús* (*khalkeús*, *nomeús*, *pompeús*; see Sect. 3.2) rather than from the -*eús* nouns themselves. These would then be transitive by virtue of having become reanalyzed (or newly formed) as Product verbs.

We already mentioned that Agent verbs in -*euō*, which were originally intransitive states, cannot be passivized. However, once -*euō* started to form transitive instrument, product and result verbs such as the ones in (43), these should in principle also allow passivization. However, at least in the 8^th^ century this use was not widespread and we only found one possible instance of passive use of an -*euō* verb, a resultative perfect passive from *hiereúō* (also *hīreúō*) ‘to sacrifice’, which, as already mentioned, is used as an agentive-transitive accomplishment verb from the very beginning of the attestation.

(45)**Figure:**
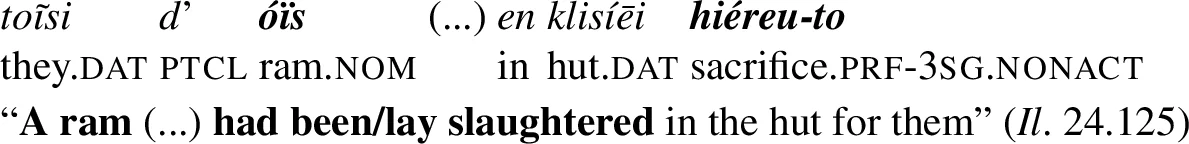

There is another possible example in Homer, given in (46), but this is usually interpreted as showing the self-benefactive use of the nonactive/middle endings rather than the passive use.

(46)**Figure:**
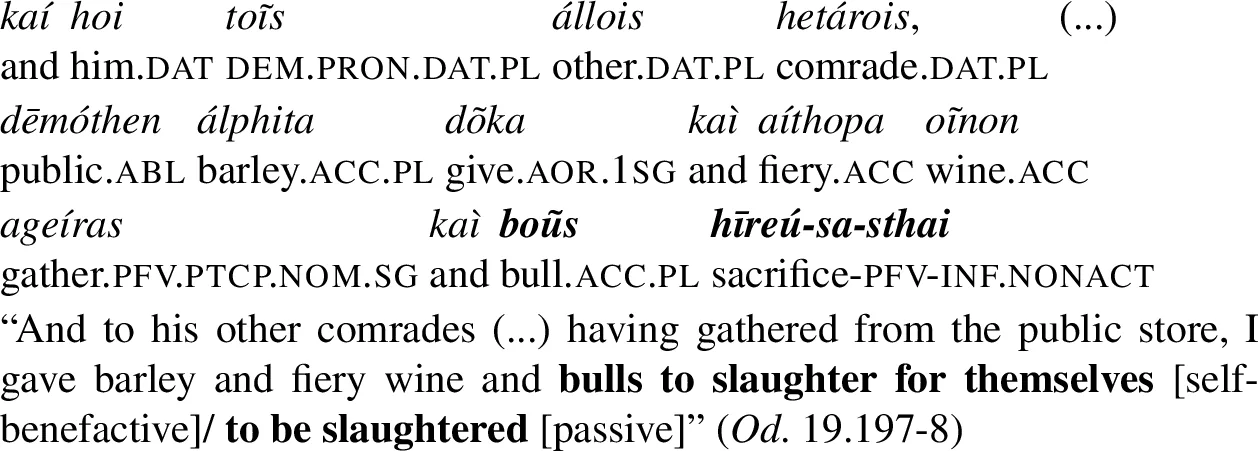

There are other instances of self-benefactive middles from transitive -*euō* verbs such as *hudreúō* ‘to draw, fetch water (mid. for oneself)’ from *húdōr* (*hudr-) ‘water’, (47), suggesting that these verbs had acquired the usual voice alternation properties of Greek transitive verbs at least in this respect and that the lack of passively used -*euō* verbs is due to the status of the accusative argument at this stage, rather than to the verbalizer itself.

(47)**Figure:**
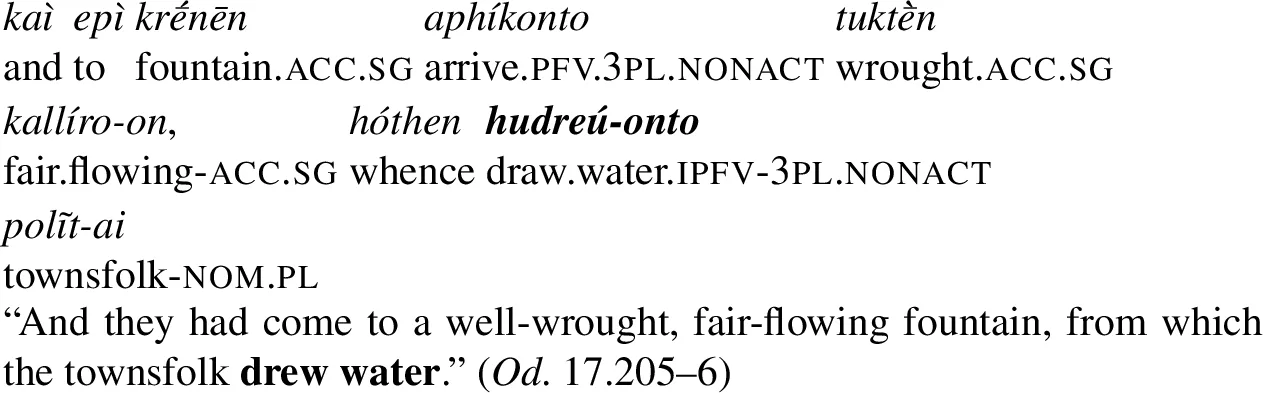

More examples of passively used -*euō* verbs, including those formed with the passive aorist suffix -*thē/e*-, occur from the 5^th^ c. BCE onward, though we forgo a more detailed analysis of this development for reasons of space. At any rate, it is clear from the 8^th^ century data that while transitivization and the self-benefactive use are already well-represented, other syntactic properties typical of agentive transitive accomplishment and activity verbs such as passivization and agent noun formation took longer to develop (or at least, to be represented in the corpus).

The latter point is crucial, because agent noun formation is one of the few available diagnostics for distinguishing unergative from unaccusative verbs in Greek, since only the former but not the latter can form agent nouns (see Grestenberger, 2018 for this and additional possible diagnostics). Agent nouns from product and instrument verbs in -*euō* are indeed already found in Homer, (48), confirming that these verbs had agent arguments (cf. Fraenkel, 1906, 212).

(48)**Figure:**
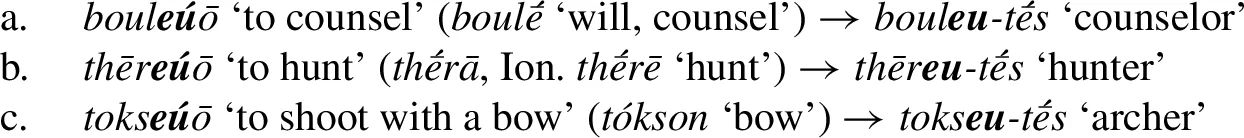

Other than *ēperop****eú****ō* ‘cheat’ → *ēperop****eu****-tḗs* ‘cheater’, no agent nouns to agent -*euō* verbs are found,^35^ but this is not surprising, given that the pseudo-agent noun that is the derivational base of such verbs was presumably semantically close enough to an agent noun to block the formation of a new one.

To summarize, by the 8^th^ century -*eu*- had been extended from its original locus — the formation of deagentive verbs from nouns in -*eu*- and other agentive nominals — to become an activity verbalizer that forms denominal verbs from instrument, product, and other bases and therefore also transitive agentive activity and accomplishment verbs. Table 11 summarizes the different structures of verbal -*eu*- and their properties in the 8^th^ c.

**Table 11:** Summary: features of verbal -*eu*-, 8^th^ c. BCE

	D-state	Activity	Activity/accompl.
features: subj.	+human, ±agent	+human, +agent	+human, +agent
features: verb	state (-dynamic)	activity (+dynamic)	activity (+dynamic)
syntax	intransitive	intransitive	transitive
ex.	*aristeúō*	*basileúō* ‘to rule’	*hiereúō* ‘to sacrifice’
	‘to be the best’	*therapeúō* ‘to serve’	*sūleúō* ‘to despoil of arms’
			*listreúō* ‘to dig with a shovel’

In the next section, we provide a formal analysis of the change from nominal to verbal -*eu*-.

## Analysis: from *n* to *v*

In this section, we propose that verbalizing -*eu*- arose from deagentive verbs derived from nouns in -*eu*-, and that their eventive readings – the precondition for a reanalysis as a *verbalizing* suffix – stem from the fact that these deagentive verbs were Davidsonian rather than Kimian states from the earliest stages of the language on.

First, we need to discuss the structure of deagentive or “pseudo-agent” verbs in the Hale & Keyser/Harley framework. Denominal verbs in this framework are formed via conflation, the definition of which is given in (49)(cf. also Hale & Keyser, 2002, 12 for a slightly different version).

(49)**Figure:**
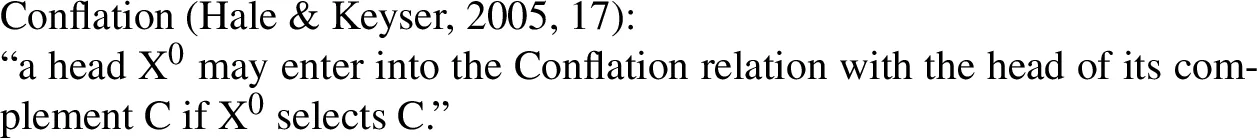

This definition basically reduces conflation contexts to head-complement relationships and crucially excludes conflation of *agents*, that is, external arguments, since these are not selected by (complements of) the relevant verbalizer, (50).

(50)**Figure:**
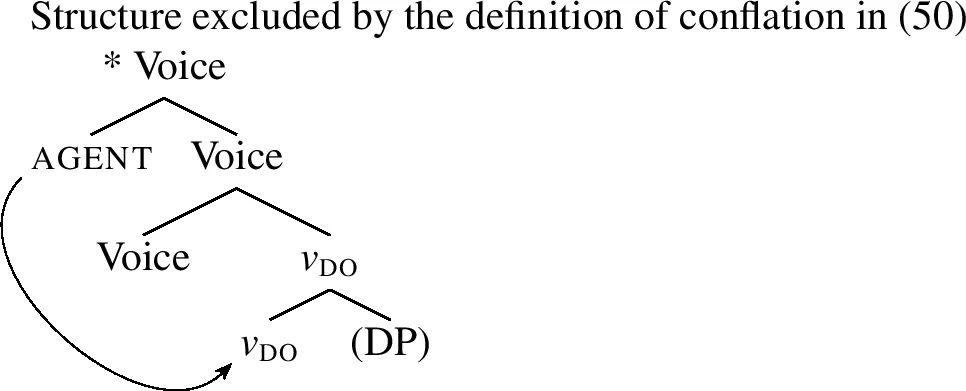

In other words, the underlying agentive noun cannot actually be the agent of the event expressed by the “deagentive” denominal verb. Nevertheless, verbs that are derived from agent nouns or nouns of profession are surprisingly common cross-linguistically, though discussed under a variety of different terms, for example “predicative” verbs (Flobert, 1975, 66; because the base noun appears to act as the predicate of the subject); “to act like *y*”-verbs (where *y* is an animate noun; Xu et al., 2007); “pseudo-agent verbs” (Bleotu, 2019, 163ff.); “behave verbs” (Oltra-Massuet & Castroviejo, 2014); “unergative behavior-related verbs” (Martin & Piñón, 2020); “manner of behaving verbs”, ‘ (Acedo-Matellán & Gibert-Sotelo, 2022; Fábregas, 2023), among others; this is the class we have called “deagentive verbs”. Examples for these verbs are given in (51)for a variety of languages.

(51)**Figure:**
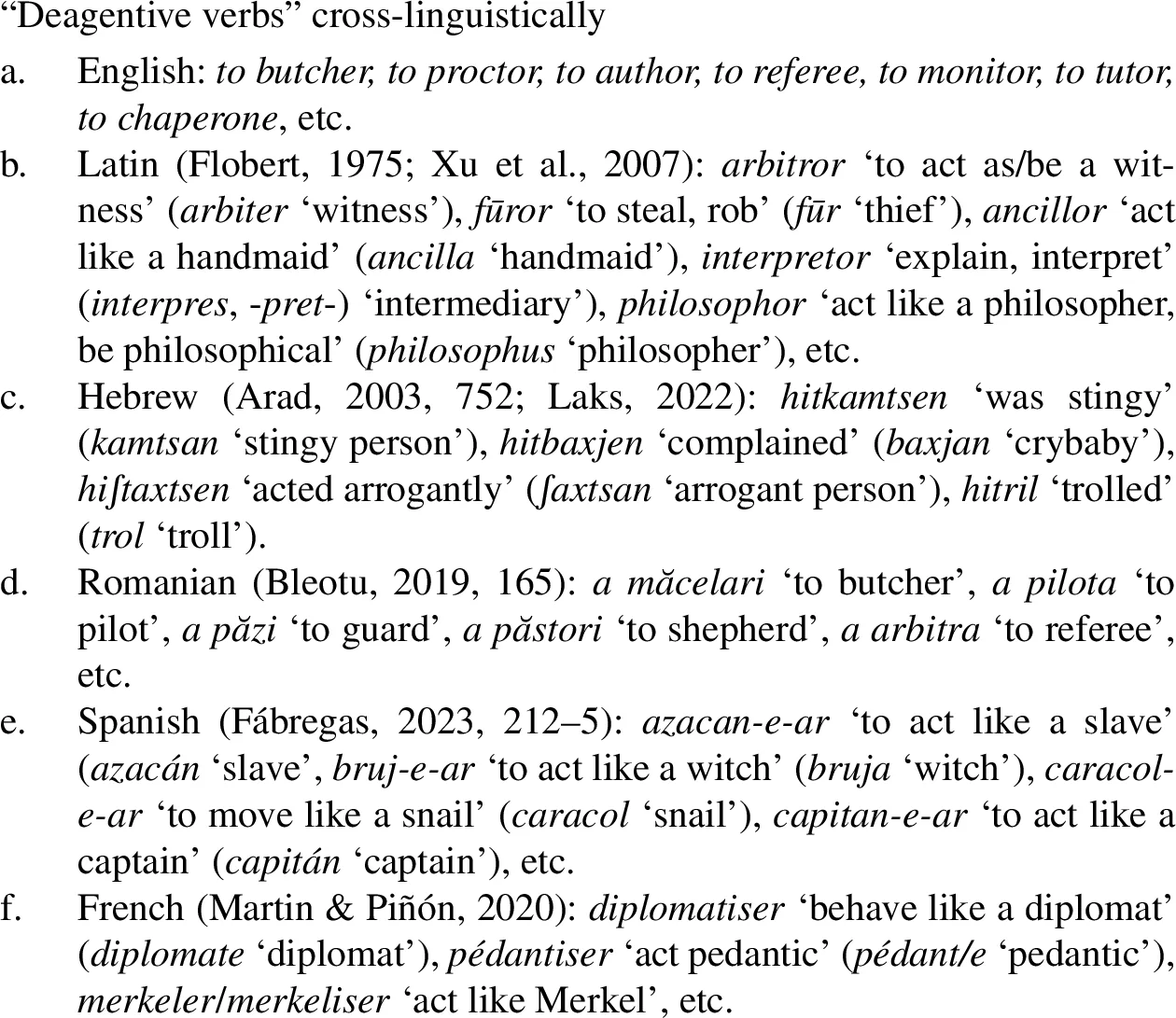

It is clear that the AG deagentive -*euō* verbs of the type *basileúō* also belong to this cross-linguistically common class. We therefore take the insights from previous analyses of these verbs as our starting point. Two points emerge as (quasi-)*communis opinio*: 1) the derivational base is generated as the complement of the selecting verbalizer in some sort of predication structure (cf. Flobert’s term *prédicatif* “predicative” for these verbs) that contains a covert preposition “like” (Bleotu, 2019) or a relational P_manner_ or other prepositional functional head (Oltra-Massuet & Castroviejo, 2014; Fábregas, 2023), in which case the (predicative) base noun would be generated in the PP complement of the verbalizer and hence fulfill the precondition for conflation, and 2) deagentive/“behavior” verbs are unergative, which in the framework pursued here means that their external argument is merged by Voice (Alexiadou et al., 2015; Grestenberger, 2018; Kastner, 2020).

The proposed structure of deagentive -*eúō* verbs is therefore as in (52). We do not see any clear arguments for or against base-generating the surface subject in the PredP together with the base noun (indicated by the trace “t_*i*_” in brackets) or merging it directly in the specifier of VoiceP. We have not yet provided detailed evidence that deagentive -*eúō* verbs are, in fact, unergative, though we discuss circumstantial evidence for this claim below.

(52)**Figure:**
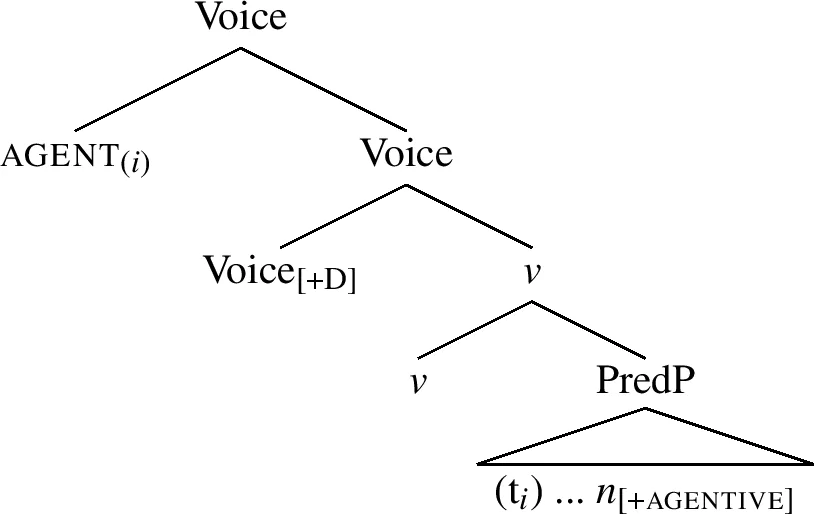

After head movement, the resulting structure would then be (53)(leaving out the higher verbal functional projections).

(53)**Figure:**
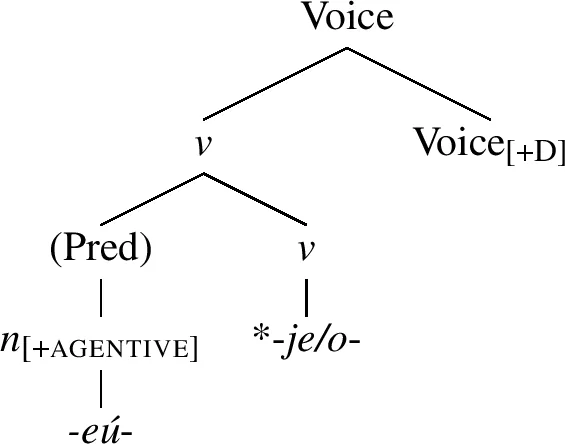

We propose that in deagentive verbs such as (53), the [+agentive] (or [+human, +animate] feature associated with *nominal* -*eu*- was reanalyzed as belonging to the selecting verbalizer through *Upwards Reanalysis*, a type of diachronic reanalysis whereby phonological and/or morphosyntactic feature(s) associated with a terminal node *x* are reanalyzed as belonging to a structurally higher, linearly adjacent head *y* (Grestenberger, 2023) within a complex word form, as illustrated in (54).

(54)**Figure:**
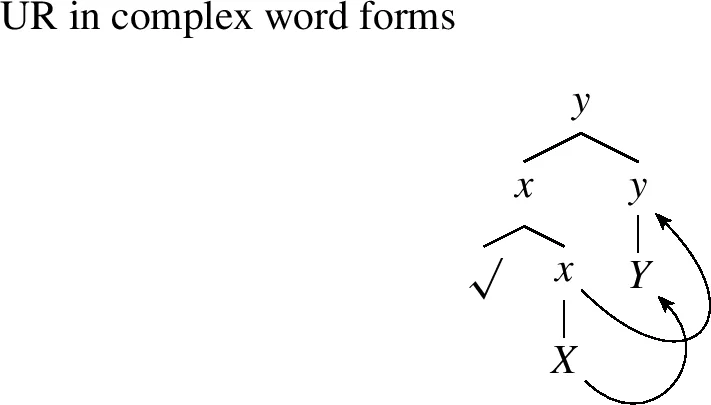

This type of reanalysis whereby “lower” (often “lexical”) elements become reanalyzed as “higher” functional elements is well known from work on syntactic change, especially in the verbal domain (cf. Roberts & Roussou, 2003; Roberts, 2010; Cournane, 2015; van Gelderen, 2018, etc.). In DM, where morphological structure in the word formation domain is expected to closely mirror syntactic structure, the same reanalysis leads to directional changes in the category and functional features of affixes, as argued by Grestenberger (2023) (see also Alexiadou, 2021; Calabrese & Grestenberger, 2024). This is exactly what happened to nominal -*eu*-, whose phonological and morphosyntactic properties (i.e., the [+agentive] feature) were transferred to the selecting verbalizer in the course of the reanalysis, illustrated in (55a) (the arrows indicate diachronic reanalysis, not movement). Together with the loss of palatal glides, this reanalysis resulted in the structure in (55b) for verbal -*eu-e/o*-, now descriptively a “complex thematic” verbalizer (cf. Grestenberger, 2022) that follows the thematic conjugation inherited from the *-*je/o*- verbalizer.

(55)**Figure:**
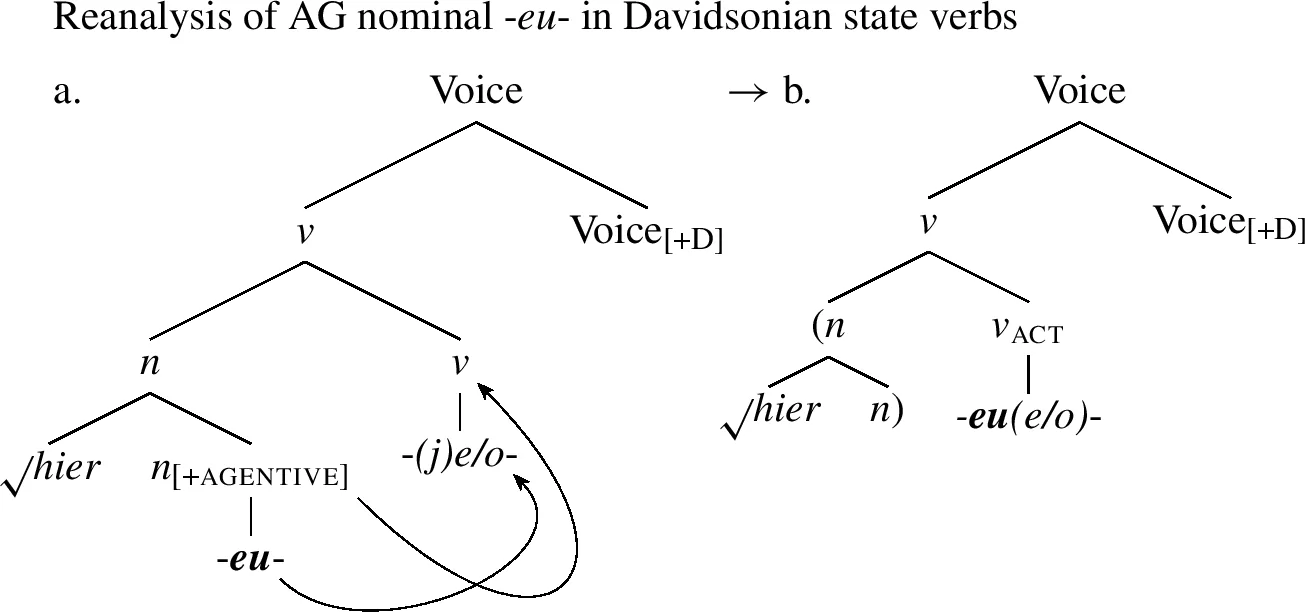

Some clarifications concerning (55)are in order. First, we are not claiming that the reanalysis in (55)created a new “flavor of *v*” — *v*_act_ — from scratch. Rather, as one anonymous reviewer points out, “*v*_act_, in the Hale and Keyser/Harley conception of types of verbalizer, is part of the Universal Grammar (UG) and it imposes the agentive animate entailments on the event it modifies by itself.” Unergative activity verbs naturally existed in AG already before the reanalysis we propose here and would receive the same decomposition into a *v*_act_ verbalizer and an external argument introduced by Voice. However, in the DM approach to morphological realization, one and the same flavor of *v* (or *n* and *a*, for that matter) can be realized with different allomorphs depending on the morphosyntactic context, in particular the root or (in secondary derivation) categorizer adjacent to the verbalizer. For example, AG has several suffixes that form alternating factitive (‘make x’)/fientive (‘become x’) change-of-state (CoS) verbs from adjectives, such as -*aíne/o*- (*leukós* ‘white, bright’ → *leuk-aíne/o*- ‘make bright’ (act.), ‘become bright’ (mid.)), -*ū́ne/o*- (*mégas*, *megal*- ‘great, big’ → *megal-ū́ne/o*- ‘make big, great’ (act.), ‘became big, great’ (mid.)), and -*oe/o*- (< *-*o-je/o*-; *orthós* ‘straight’ → *orth-óe/o*- ‘make straight, straighten’ (act.), ‘become straight’ (mid.)). Given that the syntactic and semantic properties of these verbs are otherwise uniform, it makes sense to analyze these verbalizing suffixes as (root-conditioned) allomorphs of the CoS verbalizer *v*_become_ (or *v*_cause_), (56)(we use “^⌢^” to indicate linear precedence through concatenation, following, e.g., Embick, 2015).

(56)**Figure:**
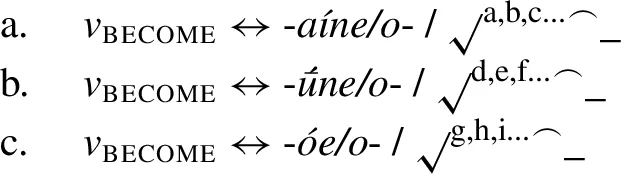

Note that the “root diacritics” used in (56)are merely used as a stand-in to indicate different groups of roots; we take no stand here on whether these are phonologically or semantically identifiable in the syntactic derivation. Moreover, the complex thematic suffixes in (56)are themselves the result of a diachronic reanalysis of a stem-final segment of the adjectival or nominal base plus the verbalizer *-*je/o*-,^36^ similarly to the reanalysis of -*eu(-)e/o*- < *-*eu-je/o*-, so their selectional properties (descriptively root- vs. stem-derived) must have changed together with their resegmentation. Crucially, both *v*_become_ and *v*_act_ gained new Vocabulary Items in the course of this resegmentation: What happened in the case of -*eu(-)e/o*- is that this became a new allomorph of the activity verbalizer *v*_act_, contextually conditioned by (specific types of?) agentive *n* (recall that most -*euō* verbs are classified as agent verbs in our corpus up to the fourth c. BCE) or specific roots (whether “nominal ones” *à la* Harley, 2005; Acquaviva, 2009 or just distinguishable by diacritics).

(57)**Figure:**
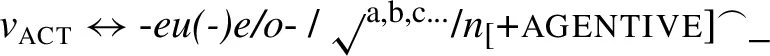

We do think that it is crucial, however, that both the phonological *and the semantic* features of the base were transferred to the verbalizer in the course of the reanalysis. That is, in structures like (55), the selecting verbalizer became explicitly identified with the flavor *v*_act_ precisely because of the specific semantics of the nominal base. This is important because as we briefly discussed in Sect. 3.1, the verbalizer *-*je/o*- itself seems to have been a default realization of different types of *v*, not associated with a particular “flavor” (see also Barber, 2013, ch. 7; Marescotti, 2024, 2026). Though there may be reason to believe that *v*_act_ itself is the default verbalizer in certain classes of denominal verbs, these do not necessarily give rise to specifically (de)agentive or exclusively intransitive activity verbs.^37^ In other words, we do not think that it is a coincidence that the specific flavor *v*_act_ emerged precisely in the deagentive -*euō* class. Rather, we think that the reanalyzed [+agentive] feature in the verbal domain was identified precisely with the verbalizer *v*_act_ as a semantic correspondent of the same feature in the nominal domain. We moreover identify this verbalizer with the intransitive unergative *v*_do_ of Hale and Keyser (1998), Hale and Keyser (2002), Hale and Keyser (2005); Harley (1999), Harley (2005); Folli and Harley (2005), Folli and Harley (2007); we use *v*_act_ for this particular “flavor of *v*” to emphasize that it builds activities. Note that the later development of verbal -*eu*- all the way to Modern Greek -*ev*-, which we briefly discussed at the beginning of Sect. 3.1, moreover suggests that this verbalizer, too, eventually became semantically bleached and was no longer associated with a particular “flavor”.

On the phonological side, a further caveat is in order: Although the loss of the palatal glide of the verbalizer *-*je/o*- coincided with the restructuring of the lexical entries of a number of denominal and deadjectival verbalizers in AG around the same time, it is difficult to say whether this sound change actually *caused* the morphological reanalysis. While “phonological erosion” can certainly facilitate resegmentation, it does not appear to be a necessary condition for morpheme reanalysis (Grestenberger, 2023; cf. also the examples of affix conglutination and telescoping in Haspelmath, 1995).

Crucially, the deagentive Davidsonian states in (55a) and their reanalyzed counterparts in (55b) are both unergatives, that is, they have an external argument merged by Voice_[+D]_ and hence an agent subject. We propose this for Ancient Greek for several reasons. First, as already mentioned, the literature on deagentive “behavior” verbs suggests that these verbs are unergatives cross-linguistically. Second, in AG, Voice_[+D]_ is the “flavor” of Voice that gives rise to canonical agentive verbs formally marked with the *active* endings, as argued by Grestenberger (2018, 2021b, 2022) based on the Voice typology of, e.g., Alexiadou et al. (2015); Schäfer (2017); Kastner (2020). This explains why the intransitive deagentive Davidsonian state *-euō* verbs always inflect with the active rather than the nonactive endings. Even though there are as of yet no uncontroversial diagnostics for unaccusative vs. unergative behavior in Ancient Greek, additional arguments can be made based on the tentative suggestions in Grestenberger (2018) and Bozzone (2020). We have already seen one argument in Sect. 5.3, namely the formation of agent nouns, which presupposes an external/agent argument and hence unergative syntax. The second potential piece of evidence concerns the formation of “object-oriented” verbal adjectives formed from the root or verbal stem with the suffix -*to*- (nom.sg.m. -*to-s*, f. -*tē*, n. -*to-n*). Grestenberger (2018) argues that these can be used as an unaccusativity test: broadly speaking, -*to*-adjectives can be formed from intransitive verbs with an underlying internal argument (that is, unaccusatives), cf. Engl. *gone*, *fallen*, etc., and to transitive verbs, in which case they are object-oriented resultatives (cf. Engl. *seen*, *beaten*, *thrown*). In other words, intransitive unergative verbs are not expected to form verbal adjectives in -*to*- and there are indeed no such verbal adjectives formed to -*eu*- verbs attested in the 8^th^ c.^38^ However, due to the complexities surrounding the actual uses of -*to*- in AG and the fact that this is an “absence of evidence” argument, this should only be taken as an observation at this point. For now, we posit the structure in (55) with an unergative Voice_[+D]_ head based on 1) the solid cross-linguistic parallels mentioned above, 2) the fact that this most easily accounts for the consistent active voice morphology of deagentive -*euō* verbs and 3) the evidence for unergative agentive syntax discussed in Sect. 5.3.

Of course, once -*eu*- has been reanalyzed as a verbalizer, it should in principle be able to combine with other types of Voice heads as well, such as nonactive Voice_[-D]_ in (often *media tantum*) experiential and speech act verbs like *bouleúomai* ‘to take counsel with oneself, deliberate’ and *manteúomai* ‘to prophesy’ and with “passive input” Voice in those cases in which transitive -*euō* verbs can be passivized (see Sect. 5.3). What we do not find, however, is any sort of Aktionsart-conditioned alternation between a stative and an activity reading that correlates with a difference in voice morphology, either at the Homeric stage or later. This suggests that the intransitive Davidsonian state reading of deagentive -*euō* and its later uses as an activity verbalizer are structurally identical at least with respect to the status of their surface subjects. In other words, the syntactic properties of *-euō* verbs — an external argument merged by Voice_[+D]_, intransitivity, and an “agentive” subject — stay the same before and after the initial reanalysis illustrated in (55). We believe that this is evidence that the Early Semantic Stability Hypothesis of Bar-Asher Siegal (2025), which states that at the initial stage of the reanalysis, the semantic change is minimal and does not affect the truth-conditions of the proposition, also holds in the domain of word structure reanalysis.

On the morphosyntactic side, this reanalysis is moreover strictly local in that it takes place between linearly adjacent and concatenated nodes, which could suggest that a diachronic analogue to the concatenation statement of Embick (2015) in (58)is at play: nodes need to be adjacent and concatenated in order for their features to be reanalyzable. In fact, together with the observation that morphosyntactic reanalysis is directional as discussed above, we can actually posit a stricter condition: in a concatenation sequence *X*^⌢^*Y*, (phonological/morphosyntactic) features of X may be transferred to Y, but not vice versa.

(58)**Figure:**
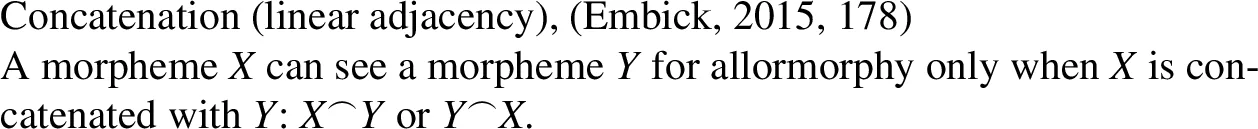

However, we take no stance as to whether this is also strictly true for all types of synchronic allomorphic interactions, some of which have been argued to be non-local or require structural rather than linear adjacency (see fn. 4 and the references cited there).

The semantic and morphosyntactic constraints (i.e., locality conditions) on reanalysis in the word formation domain therefore conspire to form a relatively constrained space of possible changes of affixes (and changes predicted to be impossible), which future work should explore.

## Conclusion

In this paper, we have argued that Homeric -*euō* verbs originally formed *Davidsonian states*, in line with their origin as denominal verbs from “agentive” animate nouns. In these verbs, the erstwhile nominal suffix -*eu*- became reanalyzed as associated with the event variable contained by definition in the denotation of D-states and hence as a verbalizer. More concretely, the features [+animate, +human] of the base noun of these (originally denominal) Davidsonian states were reanalyzed as belonging to the selecting *v*, which was identified with the activity verbalizer *v*_act_ (*v*_do_ in the Hale & Keyser/Harley framework). This led to the development of agentive activity verbs formed with verbal -*eu*- and, in a third step, the formation of transitive activity and accomplishment verbs using this suffix. This reanalysis must have taken place already before the Homeric stage: the properties of deagentive -*eu*- verbs suggest that the Davidsonian event reading is available right from the start, which is in line with cross-linguistic observations concerning the properties of “unergative behavior-related verbs” (Martin & Piñón, 2020; Acedo-Matellán, 2022; Acedo-Matellán & Gibert-Sotelo, 2022; Fábregas, 2023).

Crucially, the argument structure properties of the new verbalizer after reanalysis reflect its historic source (that is, animate nouns of profession/“agentive” nouns) in a way that reflects relatively strict locality conditions on the interaction of adjacent terminal nodes and their realizations. These properties moreover follow from the hypothesis that diachronically, this interaction is *directional*: in denominal verbs, the reanalysis of the exponence of the nominalizer *n* as that of the verbalizer *v* is a possible path of change, but the opposite direction (reanalysis of the exponence of *v* as that of *n*) is not.

Future research should investigate the syntactic and semantic behavior of -*euō* verbs in post-Classical and Byzantine Greek, which we have not been able to cover here. Specifically, it would be interesting to explore to what extent the state/event ambiguity at the heart of the development of verbal -*eu*-/-*ev*- continues to be attested across centuries. Conversely, the transitivization of this class and, connected to this, the diachronic development of the possibility of passivization also deserves further study. Moreover, the diachrony of other semantic classes of denominal and deadjectival verbs also deserves further study with respect to possible generalizations regarding their diachronic development and the grammaticalization of new Aktionsart markers from former Result, Product, etc., verb classes. For example, the development of the Ancient Greek verbalizers -*oe/o*-, -*ūne/o*-, and -*aine/o*- briefly mentioned in Sect. 6 and their extension to new adjectival and nominal bases through reanalysis of the stem-forming morpheme as part of the verbalizer suggests that certain types of deadjectival/denominal Result verbs can give rise to new factitive verbalizers (cf. Tucker, 1981, 1990; Rau, 2009, 115–120, 152–155; Villanueva Svensson, 2024 on the diachrony of these verbs). This development thus instantiates the same *n* > *v* reanalysis as the one we discussed in this paper. Future research will undoubtedly unearth more such regular morphosemantic changes in the event and argument structure domain.

## Data Availability

All data supporting the findings of this study are included within the paper and its Appendix. The datasets are available for download at https://zenodo.org/records/23337517.

## Appendix: Homeric verbs in -*euō*

**Table 12:** Homeric -*euō* verbs

Verb	Meaning	Derivational base	Sem. class	-*eus* noun	(in)tr.
*agoreúō*	speak in the assembly	*agorá* ‘assembly, gathering’	Location	no	tr.
*akheúō*	grieve	*ákhos* ‘grieve, pain’	Source	no	intr.

^a^A poetic variant of *dīneúō* ‘to whirl, spin around’ according to Fraenkel (1906, 186) and Nikolaev (2025), itself a Product verb from *dī́nē*.

^b^See Nikolaev (2023, 7).

Table 12 lists the Homeric -*euō* verbs that form the basis of our study, their meaning, their derivational base, the semantic class we assigned to each verb (see Sect. 4.3), whether or not a contemporary noun in -*eu*- exists (usually the derivational base, if yes), and the transitivity of the verb (intr./tr.). To determine the derivational base, we relied on the relevant etymological dictionaries (Frisk, 1960–1972; Chantraine, 1968–1980; Beekes, 2010). However, because the focus is on the *synchronic* analysis that would have been available to speakers at the time, we also relied on synchronic criteria such as formal and semantic transparency (see Grestenberger & Kastner, 2022 for discussion). This means that for some entries, more than one analysis is available, and the most obvious synchronic analysis may not correspond to the etymological provenance (as in the case of *manteúomai*, semantically associated with *mántis* ‘seer, prophet’, but morphologically based on unattested **manteús* or **mántēs*).

## Footnotes

1 The history of Greek is conventionally divided into the following periods (cf. Horrocks, 2010; Pantelidis, 2026): **Proto-Greek** (reconstructed), 3rd millennium BCE; **Mycenaean Greek**, 14th – 12th century BCE; the **Dark Ages** (no written records), 13th – 8th century BCE; **Ancient Greek**, 8th – 4th century BCE; **Koine Greek**, 4th century BCE – 4th century CE; **Medieval Greek**, 4th – 15th century CE; **Early Modern Greek**, 15th – 19th century CE; **Modern Greek**, 19th century CE to the present. The Mycenaean Greek corpus consists of very short domestic texts attested almost exclusively on clay tablets, while the earliest continuous literary texts are the Homeric poems, dated to the 8th century BCE. The present study focuses on Homeric Greek.

2 In the following, we gloss the complex °*eu-e/o*- as v.ipfv since it synchronically behaves as a complex thematic imperfective verbalizer (Grestenberger, 2022). For reasons of consistency, we also do this in cases in which it is likely that the -*eu*- part of the suffix was still synchronically nominal; this will be made clear from the discussion and the gloss of the nominal base. The transliteration system used here is as follows: α = *a*, β = *b*, γ = *g*, δ = *d*, ε = *e*, ζ = *z*, η = *ē*, θ = *th*, ι = *i*, κ = *k*, λ = *l*, μ = *m*, ν = *n*, ξ= *ks*, ο = *o*, π = *p*, ρ = *r*, σ/ς = *s*, τ = *t*, υ = *u*, φ = *ph*, χ = *kh*, ψ = *ps*, ω = *ō*; ῾ = *h*, ει = *ei*, ου = *ou*, ῆ = *ẽ*, ῶ = *õ*.

3 See Borer (2013, 2014) for criticism and Dahl and Fábregas (2018); Grestenberger and Kastner (2022) for a defense of zero categorizers.

4 The locality conditions on allomorphy and in particular the question of structural vs. linear adjacency conditions are subject to debate, see, for example, Carstairs (1987), Bobaljik (2000), Embick (2010, 2012, 2015), Merchant (2015), Moskal and Smith (2016), Paparounas (2024), Angelopoulos and Spyropoulos (2025), Kalin (2026); the papers in Gribanova and Shih (2017) and Kastner and Moskal (2018), among many others. We cannot address this debate here, but the reanalysis we argue for in Sect. 6 crucially depends on interactions between the exponents of linearly adjacent nodes. Whether this is generally the case in diachronic reanalysis of exponence is a matter for future research.

5 The *v*+Voice+Asp node can be phonologically empty under certain circumstances which do not concern us here. T is realized as an antitropal prefix in the past tenses. See Grestenberger (2022) and Calabrese and Petrosino (2023) for a more detailed discussion of the morphological structure of the Ancient Greek verb.

6 Or a “nominal root”, cf. Harley (2005), Bleotu (2019).

7 An anonymous reviewer noted that MG nouns in -*ea*-, such as MG *ippéas* ‘horserider’, are historically derived from the reanalysis of the accusative singular of nouns in -*eu*- as a stem-forming suffix. From this perspective, MG verbs in -*ev-o* are historically also based on nominal stems in -*eu*-, although their relationship has become opaque.

8 “Complex thematic suffixes” such as *-*je/o*- minimally consist of (an) initial consonant(s) plus the “theme vowel” with its two allomorphs -*e*- and -*o*-, hence *-*j(-)e/o*-, -*sk(-)e/o*-, -*n(-)e/o*-, -*az(-)e/o*-, *-*ej(-)e/o*-, etc. (see Grestenberger, 2022 on AG complex thematic suffixes; the synchronic distribution of the theme vowel allomorphs is described in Smyth & Messing, 1956, 110–118; van Emde Boas et al., 2019, 114–118).

10 See Grestenberger (2022, 31) for arguments that *-*je/o*- and -*s(a)*- should both be treated as primary verbalizers.

11 Like in the verbal system, the *nominal* theme vowel -*o*- also had an allomorph -*e*-, though this has largely been eliminated from the synchronic inflectional paradigm (remnants remain in the vocative, e.g., *ánthrōp-e* ‘O human!’ and in some archaic locative forms). However, it is still visible as the basis of various secondary derivations, among them precisely AG -*e(-)u*- (see Schindler, 1976, 351–352).

12 Thus already Schindler (1976, 350–351), who also discusses other examples of descriptive synchronic replacement of the nominal theme vowel by derivational suffixes in secondary (i.e., stem-based) derivation in Greek; cf. also Schindler (1980); Nussbaum (1999, 2014); Fellner and Grestenberger (2016); Grestenberger (2017, 2021a), a.m.o. on this replacement pattern in Greek and other IE languages.

15 https://stephanus.tlg.uci.edu/.

16 https://figshare.com/articles/dataset/The_Diorisis_Ancient_Greek_Corpus/6187256/1.

17 We are grateful to Hannes Fellner for his help with the DIORISIS corpus.

18 In Homer, some of these forms have been argued to replace their verbal bases for “rhythmical” (metrical) reasons, cf. Chantraine (1948, 368); on the relationship between denominal and primary verbs in -*éō* and those in -*eúō* see also Fraenkel (1906, 205–208), Tucker (1990, 80–81, 116–122), Nikolaev (2025, 114–115).

19 An alternative analysis is also possible, whereby **hagistós* ‘most holy’ is taken as the derivational base of *hagisteúō*, while *hagisteía* is interpreted as a secondary derivative of the verb, in accordance with the productive morphological pattern -*euō* : -*eía* (cf. *basileúō* : *basileía*). This interpretation provides a plausible account of the morphological relationships among these forms.

20 The derivation of *nukheúō* from the word for ‘night’, *núks*, *nukt*-, is not unproblematic. The absence of a *-t-* and presence of an aspirate in the verbal stem has long been recognized as a morphological difficulty, suggesting that the verb is actually based on a stem allomorph *nukh*- (cf. Hsch. νύχα· νύκτωρ *núkha*: *núktōr* adv. ‘at night’), which subsequently served as the immediate derivational base of *nukheúō* (see Beekes, 2010, 1027 for discussion and references).

22 The derived feminine of *basileús* ‘king’ is *basíleia* (< **basíleu-ja*) ‘queen’. Denominal verbs can in principle be formed from *-*ja* stems, for example, *peĩra* (< **pér-ja*) ‘test, attempt’ → *peiráo-mai*, -*ō* (< **per-já-je/o-*) ‘to attempt’ (cf. Tucker, 1990, 244), and the -*euō* verb *aletreú-ō* ‘to be/act as a female corn-grinder; to grind corn’ from *aletr-ís* ‘female corn grinder’ shows that denominal verbs could in principle take derived feminine nominal stems as their basis. This suggests that the use of *basileúō* in reference to a woman in *Il*. 6.426 is not due to a morphological restriction.

23 Though Fraenkel (1906, 221) notes that -*euō* verbs in the Attic varieties of the 5^th^ and 4^th^ c. BCE and later on in Koine Greek usually take the middle endings “wofür wir das Aktiv erwarten” (“where we would expect the active”), so this could suggest that these variants did indeed treat the surface subject of these verbs differently, namely as holders or undergoers. However, this issue requires further study.

24 On apparent counterexamples of manner and locative modification of K-states see also Maienborn (2007); for criticism of the frame-setting explanation see Ernst (2016).

27 E.g., *Know your audience!*, *Be more English!*, *I’m loving it*, etc., see, e.g., De Swart (1998) on aspectual coercion.

28 This could also be a (metrical?) replacement of synonymous *pontoporéō*, the morphologically expected denominal *-*je/o*-verb to a (compounded) thematic *o*-stem (Tucker, 1990, 76, 80–81). We include it here and in the Appendix because it is an Agent verb either way.

29 See Maienborn (2001, 2019) for details.

30 See footnote 25.

31 We are grateful to Jonathan Bobaljik for suggesting this comparison to us.

32 The etymological dictionaries (Frisk, 1960–1972; Chantraine, 1968–1980; Beekes, 2010) treat the -*eu*-noun as a backformation from the verb, but purely because of the relative frequency of attestation (noun 1x in Homer vs. verb 7x in Homer and once in the Homeric hymns), which is not a linguistic argument. Semantically, there is nothing wrong with treating *ēperopeúō* as an Agent verb. The nominal derivational basis of *ēperopeús* itself is, however, unclear.

33 They can become nominative subjects under passivization from the 5^th^ century onward, though, as can dative and genitive objects (cf. Schwyzer, 1950, 240–1; Anagnostopoulou & Sevdali, 2015, etc.). This could suggest a change in the case system around that time.

34 An anonymous reviewer raises the question whether the aorist suffix -*s(a)*- played a role in the transitivization of the -*euō* class, since -*s(a)*- is specifically associated with transitive perfectives, whereas intransitive perfectives tend to take -*th*- (including in denominal and deadjectival verbs, cf. Tucker, 1990, 297–316). However, we do not think that the aorist suffix played a role in the transitivization process. First, out of our 53 derived -*euō* verbs in Homer, only 17 occur with -*s(a)*-aorists (*agoreúō* ‘speak in the assembly’, *basileúō* ‘to be king’, *bouleúō* ‘take counsel’, *daitreúō* ‘to divide’, *ēperopúō* ‘to cheat’, *hēgemoneúō* ‘to lead’, *hiereúō* ‘to sacrifice’, *hiketeúō* ‘to be a suppliant; beseech’, *keleúō* ‘to urge, drive on’, *litaneúō* ‘to pray, entreat’, *manteúomai* ‘to divine, prophesy’, *mnēsteúō* ‘to court, seek in marriage’, *oisteúō* ‘to shoot arrows’, *opīpeúō* ‘to look at, stare at’, *phuteúō* ‘to plant’, *thēteúō* ‘to be a serf’, *tolupeúō* ‘to wind wool/yarn into a clew’), and most of these aorist stems are attested only once or twice compared to the far better attested imperfective *-*je/o*- stem (out of the ca. 800 tokens of -*euō* verbs in Homer, 540 are imperfective forms whereas only 210 are perfective -*s(a)*-stems; the rest are from the perfect or future stems). The majority of the perfective forms moreover belongs to only a couple of verbs; 59 -*s(a)*-aorist forms alone belong to *keleúō*, which is probably an “extension” of primary *kelé/ó*- (we classify this type as activity in the Appendix; basically as deverbal). 27 forms belong to *agoreúō*, a transitive Location verb, 14 to *hiereúō*, a transitive Product verb (or maybe originally an Agent verb), and 13 to *bouleúō*, likewise a product verb, so the fact that these form transitive -*s(a)*-aorists is precisely *because* their imperfective stems are transitive in the first place. Note that the few lonely -*s(a)*-aorist forms of intransitive Agent -*eúō* verbs, including the sole aorist forms of *basileúō* in *Od*. 1.401 (usually interpreted as aorist subjunctive, but note that it could also be from the future stem) and of *thēteúō* in *Il*. 21.441, are intransitive like the corresponding imperfective stem, which is what we would expect if both *-*je/o*- and -*s(a)*- could be used as primary verbalizers (see fn. 10 and the discussion preceding it in the main text). We therefore do not think that perfective morphology played a role in the ongoing transitivization of the -*eúō* verbalizer. Rather, it followed from the argument structure properties of the different semantic classes of verbs it formed and their relationship to their nominal base(s), as outlined in the main text.

35 With the possible exception of *arneutḗr* ‘acrobat’, for which no base verb is attested (4^th^ c. verb forms in Lycophron are backformations). Fraenkel (1910, 21) points out that this must be an old form because agent nouns formed synchronically from denominal verbs otherwise use the productive agent noun forming suffix *-tēs* rather than inherited *-tēr* or *-tōr*. Peters (1993, 386–90) argues that the expected verb **arneúō* from which this agent noun was formed was itself derived from the -*eu*- noun underlying *arneiós* ‘ram’ < , whence denominal  ‘to act like a ram, to jump around like a ram’ — in other words, a deagentive -*euō* verb. Once the derivational relationship between **arneúō* ‘jump, tumble’ and *arneiós* ‘ram’ had become opaque, a new agent noun *arneutḗr* ‘tumbler, acrobat’ could thus be formed. This form is therefore precious additional (albeit indirect) evidence that the deagentive -*euō* type did indeed have agentive eventive/activity readings very early on in Greek and was thus compatible with agent noun formation. We are grateful to Alexander Nikolaev for discussion of these forms.

36 See Tucker (1981, 1990) and Villanueva Svensson (2024) on the diachrony of these suffixes.

37 See Oltra-Massuet (1999) and Bertocci (2017) for proposals that the Catalan and Latin first conjugations could be interpreted as default realization of (admittedly very different types of) *v*/Voice.

38 See Dalpedri (2025) for a more detailed description of the Homeric uses of deverbal -*to*-, confirming the fundamentally “theme-oriented” nature of this suffix.
